# Arginine methylation-dependent TRIM47 stability mediated by CARM1 promotes the metastasis of hepatocellular carcinoma

**DOI:** 10.1038/s41420-024-02244-4

**Published:** 2024-11-20

**Authors:** Yuzhe Tang, Xiang Meng, Xia Luo, Wentao Yao, Li Tian, Zijian Zhang, Yuan Zhao, Juan Xiao, Haichuan Zhu, Jia Hu

**Affiliations:** 1 Institute of Biology and Medicine, College of Life and Health Sciences, Wuhan University of Science and Technology, Wuhan, China; 2 Department of Laboratory Medicine, Tongji Hospital, Tongji Medical College, Huazhong University of Science and Technology, Wuhan, China; 3 Institute of Neuroscience and Brain Diseases, Xiangyang Central Hospital, Affiliated Hospital of Hubei University of Arts and Science, Xiangyang, Hubei China

**Keywords:** Oncogene proteins, Metastasis

## Abstract

The tripartite motif (TRIM) protein family has been shown to play important roles in the occurrence and development of various tumors. However, the biological functions of TRIM47 and its regulatory mechanism in hepatocellular carcinoma (HCC) remain unexplored. Here, we showed that TRIM47 was upregulated in HCC tissues compared with adjacent normal tissues, especially at advanced stages, and associated with poor prognosis in HCC patients. Functional studies demonstrated that TRIM47 enhanced the migration and invasion ability of HCC cells in vitro and in vivo. Mechanistically, TRIM47 promotes HCC metastasis through interacting with SNAI1 and inhibiting its degradation by proteasome. Moreover, TRIM47 was di-methylated by CARM1 at its arginine 210 (R210) and arginine 582 (R582), which protected TRIM47 from the ubiquitination and degradation mediated by E3 ubiquitin ligase complex CRL4^CRBN^. Collectively, our study reveals a pro-metastasis role of TRIM47 in HCC, unveils a unique mechanism controlling TRIM47 stability by CARM1 mediated arginine methylation, and highlights the role of the CARM1-CRL4^CRBN^-TRIM47-SNAI1 axis in HCC metastasis. This work may provide potential therapeutic targets for metastatic HCC treatment.

## Introduction

Tripartite motif-containing (TRIM) family proteins are a group of E3 ubiquitin ligases (over 80 members) characterized by a RING domain in N-terminal, a B-box domain, a coiled-coil domain, and a variable C-terminal domain. TRIM family members are involved in regulating various biological processes, such as autophagy, cell cycle and innate immune response [1]. Dysregulation of TRIM proteins is closely associated with tumorigenesis and tumor progression [2]. TRIM47, also known as GOA (Gene overexpressed in astrocytoma protein), was upregulated and possessed oncogenic function in multiple types of tumors, such as non-small cell lung carcinoma (NSCLC) [3], colorectal cancer (CRC) [4] and prostate cancer (PC) [5]. Hepatocellular carcinoma (HCC) is the sixth most common cancer and the fourth leading cause of cancer-related mortality worldwide [6]. A bioinformatics study reported that TRIM47 expression was associated with poor prognosis of HCC. A TRIM family gene-based signature including TRIM47 and another 5 TRIM genes performed well in Overall survival (OS) prediction for HCC [7]. These studies indicated that TRIM47 might play an important role in HCC progression. However, the detailed roles of TRIM47 and its regulatory mechanism in HCC remain elusive.

The transfer of methyl groups from S-adenosylmethionine (SAM) to the guanidino nitrogen atoms of arginine is catalyzed by a group of enzymes known as protein arginine methyltransferases (PRMTs), a process referred to as arginine methylation [8]. The PRMT family consists of nine members (PRMT1-9) and can be categorized into three groups based on their catalytic activity. Type I (PRMT1-4, PRMT6, PRMT8) are responsible for the formation of monomethyl-arginine (MMA) and asymmetric dimethylarginine (ADMA). Type II (PRMT5 and PRMT9) catalyze the synthesis of MMA and symmetric dimethylarginine (SDMA). Type III PRMTs are only involved in the enzymatic synthesis of MMA. Coactivator-associated arginine methyltransferase 1 (CARM1), a type I PRMTs, is deregulated in numerous cancers and plays critical roles in cancer progression by catalyzing asymmetric di-methylation of histone or nonhistone substrate proteins [9]. For example, CARM1 promoted breast cancer metastasis by methylating chromatin remodeling factor BAF155 [10]. Arginine methylation of MDH1 by CARM1 inhibits glutamine metabolism and suppresses pancreatic ductal adenocarcinoma (PDAC) progression [11]. However, independent studies reported opposite functional roles of CARM1 in HCC. CARM1 was previously reported to suppress the glycolysis in liver cancer cells by mediating arginine 234 (R234) methylation of GAPDH, thus inhibiting the proliferation of liver cancer cells [12]. This conflicts with another study demonstrating that CARM1 indicates poor prognosis and promotes HCC progression by activating AKT/mTOR signaling [13]. These conflicting results lead to a confused understanding of CARM1’s functions in HCC, thus needing to explore its underlying molecular mechanism.

Here, we found that TRIM47 was upregulated in tumor tissues and associated with poor clinical outcomes of HCC patients. In vitro and in vivo studies revealed that TRIM47 facilitated the migration and invasion of HCC cells. Mechanistically, TRIM47 promoted epithelial-mesenchymal transition (EMT) by interacting with SNAI1 and maintained its stability. Moreover, we identified that TRIM47 was a novel substrate of CARM1, while the methylation by CARM1 protected TRIM47 from proteasomal degradation mediated by E3 ubiquitin ligase complex CRL4^CRBN^. This work may provide a basis for finding novel targets and designing therapeutic strategies against metastatic HCC.

## Results

### TRIM47 is a pro-metastatic factor in HCC

**Fig. 1:**
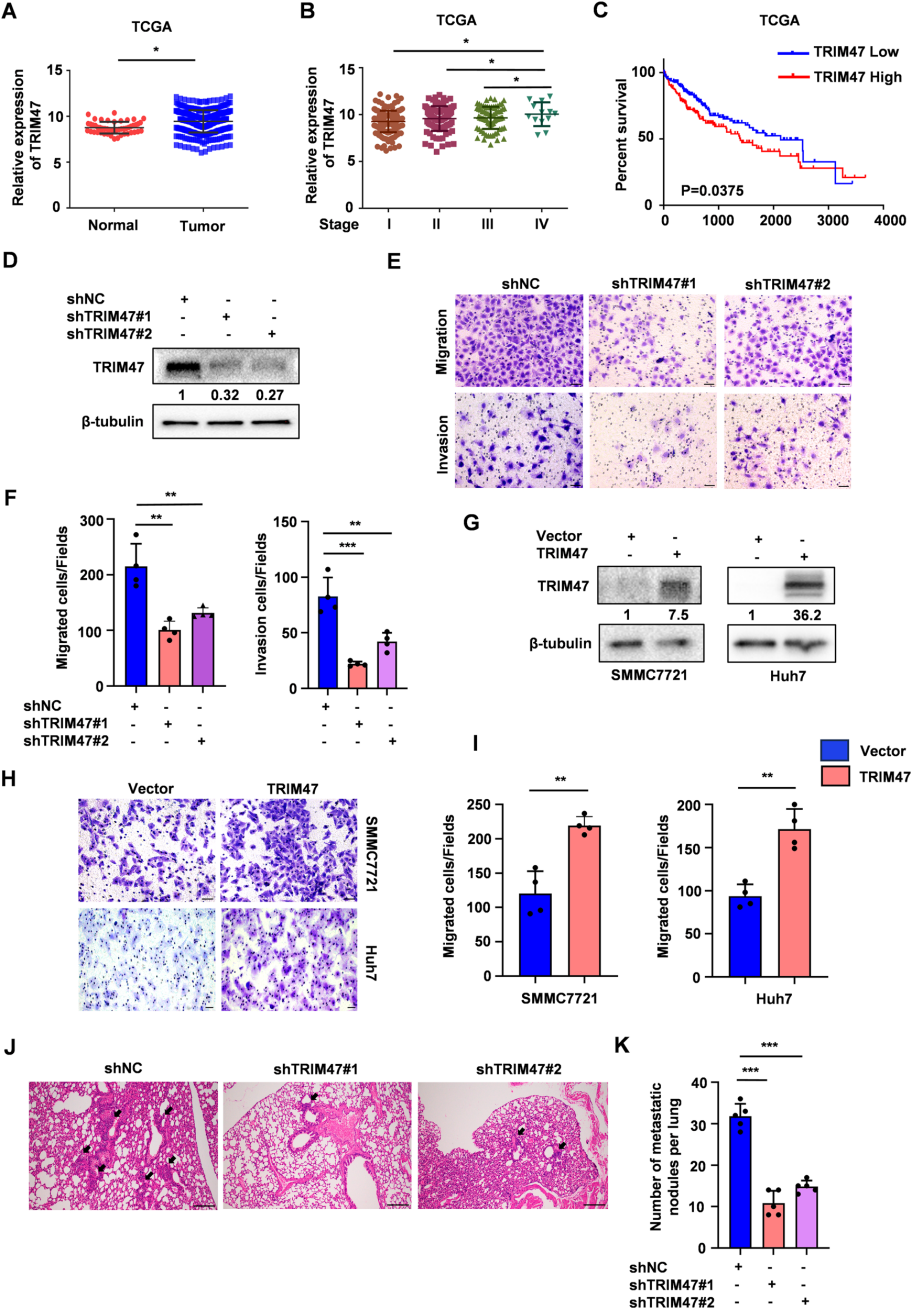
TRIM47 promotes HCC cells migration and invasion*.* **A** Analysis of TRIM47 mRNA expression in HCC primary tumor tissues and normal liver tissues from TCGA database. *P < 0.05. **B** Analysis of TRIM47 mRNA expression in different tumor stages of HCC patients from TCGA database. *P < 0.05. **C** Kaplan–Meier survival analysis of overall survival (OS) stratified by TRIM47 expression in HCC tumor tissues from TCGA database. *P* = 0.0375. **D** Western blot analysis of TRIM47 protein levels in TRIM47 stably knockdown (shTRIM47#1 and shTRIM47#2) or control (shNC) SMMC7721 cells. **E**, **F** The migration assay (upper panel) and invasion assay (bottom panel) of SMMC7721 cells with TRIM47 stably knockdown (**E**). The average number of cells per field were calculated (**F**). Scale bars, 50 μm. Data are shown as mean ± SD. *n* = 3 samples per group, four fields per sample. **P < 0.01, ***P < 0.001. **G** Western blot analysis of TRIM47 protein levels in SMMC7721 cells transiently transfected with Vector or FLAG-TRIM47. **H**, **I** The migration assay of SMMC7721 cells transiently transfected with Vector or FLAG-TRIM47 (**H**). The average number of cells per field were calculated (**I**). Scale bars, 50 μm. Data are shown as mean ± SD. *n* = 3 samples per group, four fields per sample. **, *P* < 0.01. **J**, **K** 2 × 10^6^ TRIM47 stably knockdown (shTRIM47#1 and shTRIM47#2) or control (shNC) SMMC7721 cells were injected into nude mice (*n* = 5 per group) via tail vain. The representative haematoxylin and eosin (H&E) images (**J**) and quantification of metastatic foci in lungs (**K**) were shown. Black arrows indicated the metastatic foci. Scale bars, 100 μm. Data are shown as mean ± SD. ***P < 0.001.

To evaluate the clinical significance of TRIM47 in human HCC, we analyzed the publicly available HCC expression profiles in The Cancer Genome Atlas (TCGA) and GSE76427 database. TRIM47 was upregulated in HCC tumor tissues compared with normal tissues, especially at advanced stages (stage IV vs stage I/II/III in TCGA) (Fig. 1A, B, Fig. S1A). Kaplan-Meier survival analysis revealed that HCC patients with high TRIM47 levels usually had poorer overall survival (OS) (Figs. 1C and S1B). These data suggest that TRIM47 may possess oncogenic activity in HCC.

Next, we explored the function of TRIM47 in HCC progression. We detected TRIM47 expression in four HCC cell lines (HepG2, Huh7, SMMC7721 and Bel7402) and found that SMMC7721 cells expressed TRIM47 at a relatively high level while Huh7 and Bel7402 cells at a low level (Fig. S1C). SMMC7721, HepG2 and Huh7 cell lines were thus chosen for further studies. Knockdown of TRIM47 in SMMC7721 and HepG2 cells significantly inhibited cell migration and invasion in vitro (Figs. 1D–F and S1D–H). Conversely, TRIM47 overexpression promoted cell migration ability in Huh7, HepG2 and SMMC7721 cells (Figs. 1G–I and S1I–K). Moreover, TRIM47 knockdown decreased the foci number of lung metastasis in vivo (Fig. 1J–K). These findings indicate that TRIM47 is a pro-metastatic factor in HCC.

### CARM1 interacts with TRIM47 and maintains its stabilization

**Fig. 2:**
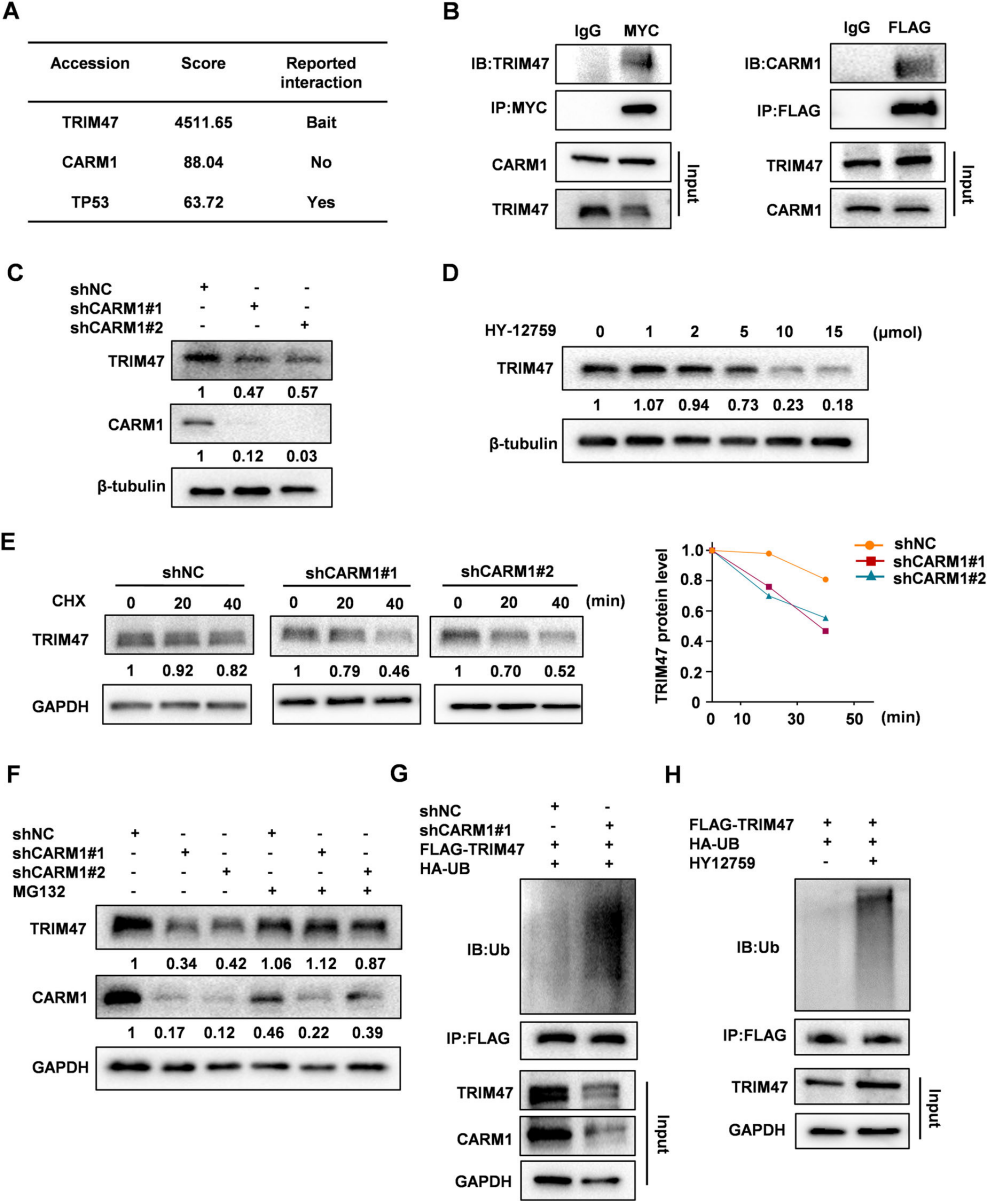
CARM1 is a binding partner of TRIM47. **A** HEK293T cells were transfected with FLAG-TRIM47 for 48 h. Cell extracts were subjected to immunoprecipitated with anti-FLAG antibody or a control IgG. CARM1 was identified via mass spectrometry. **B** HEK293T cells were co-transfected with FLAG-TRIM47 and MYC-CARM1 for 48 h. Total cell extracts were immunoprecipitated with anti-FLAG or anti-MYC antibodies. FLAG-TRIM47 and MYC-CARM1 were detected by western blot. **C** Western blot analysis of TRIM47 protein levels in CARM1 stably knockdown SMMC7721 cells. **D** Western blot analysis of TRIM47 expression in SMMC7721 cells treated with CARM1 inhibitor HY12759 for the indicated times. **E** Cycloheximide chase analysis of TRIM47 degradation in CARM1 stably knockdown SMMC7721 cells. The relative band intensity of TRIM47 was quantified and plotted. **F** Western blot analysis of TRIM47 protein levels in CARM1 stably knockdown (shCARM1#1 and shCARM1#2) or control (shNC) SMMC7721 cells treated with DMSO or 10 μM MG132 for 4 h. **G** CARM1 stably knockdown (shCARM1#1) or control (shNC) SMMC7721 cells were co-transfected with FLAG-TRIM47 and HA-ubiquitin plasmids for 48 h. Total cell extracts were immunoprecipitated with anti-FLAG antibody. The immunoprecipitates were detected with anti-ubiquitin and anti-FLAG antibodies. **H** SMMC7721 cells were transfected with FLAG-TRIM47 plasmid for 48 h and treated with HY12759 for another 8 h. Total cell extracts were immunoprecipitated with anti-FLAG antibody. The immunoprecipitates were detected with anti-ubiquitin and anti-FLAG antibodies.

To dissect the upstream regulatory factors and pro-metastatic mechanism of TRIM47 in HCC, we performed an immunoprecipitation-coupled mass spectrometry screen. CARM1, a type I PRMTs playing critical roles in cancer progression by methylating histone or nonhistone substrates, was identified as a putative TRIM47-interacting protein (Fig. 2A, Fig. S2A). Noticeably, TP53, another protein reported to interact with TRIM47 [14], was also hitted in our screening, indicating the reliability of our screening. The binding between TRIM47 and CARM1 were confirmed by reciprocal co-IP assay (Fig. 2B). Next, we determined whether the expression of CARM1 or TRIM47 was affected by their interactions. Knockdown of TRIM47 had no effect on CARM1 protein levels (Fig. S2B). However, TRIM47 protein levels was significantly decreased upon CARM1 depletion, while its mRNA expression was unchanged (Fig. 2C, Fig. S2C). Treatment with specific CARM1 methyltransferase inhibitor HY-12759 also reduced TRIM47 protein levels in a dose-dependent manner (Fig. 2D). The cycloheximide chase assay showed that the protein half-life of TRIM47 was remarkably shortened after CARM1 knockdown (Fig. 2E). These results suggest that CARM1 acts upstream of TRIM47 to control TRIM47’s protein stability through post-translational modifications. Of note, decreased TRIM47 protein levels by CARM1 knockdown were rescued when treated HCC cells with proteasome inhibitor MG132, but not with lysosome inhibitor CQ (Figs. 2F and S2D), suggesting that CARM1 protects TRIM47 protein from proteasome-mediated degradation. Consistently, the ubiquitination of TRIM47 was enhanced by CARM1 knockdown or HY-12759 treatment (Fig. 2G, H). Taken together, these results indicate that CARM1 stabilizes TRIM47 protein by inhibiting its ubiquitination.

### TRIM47 is an arginine methylation substrate of CARM1

**Fig. 3:**
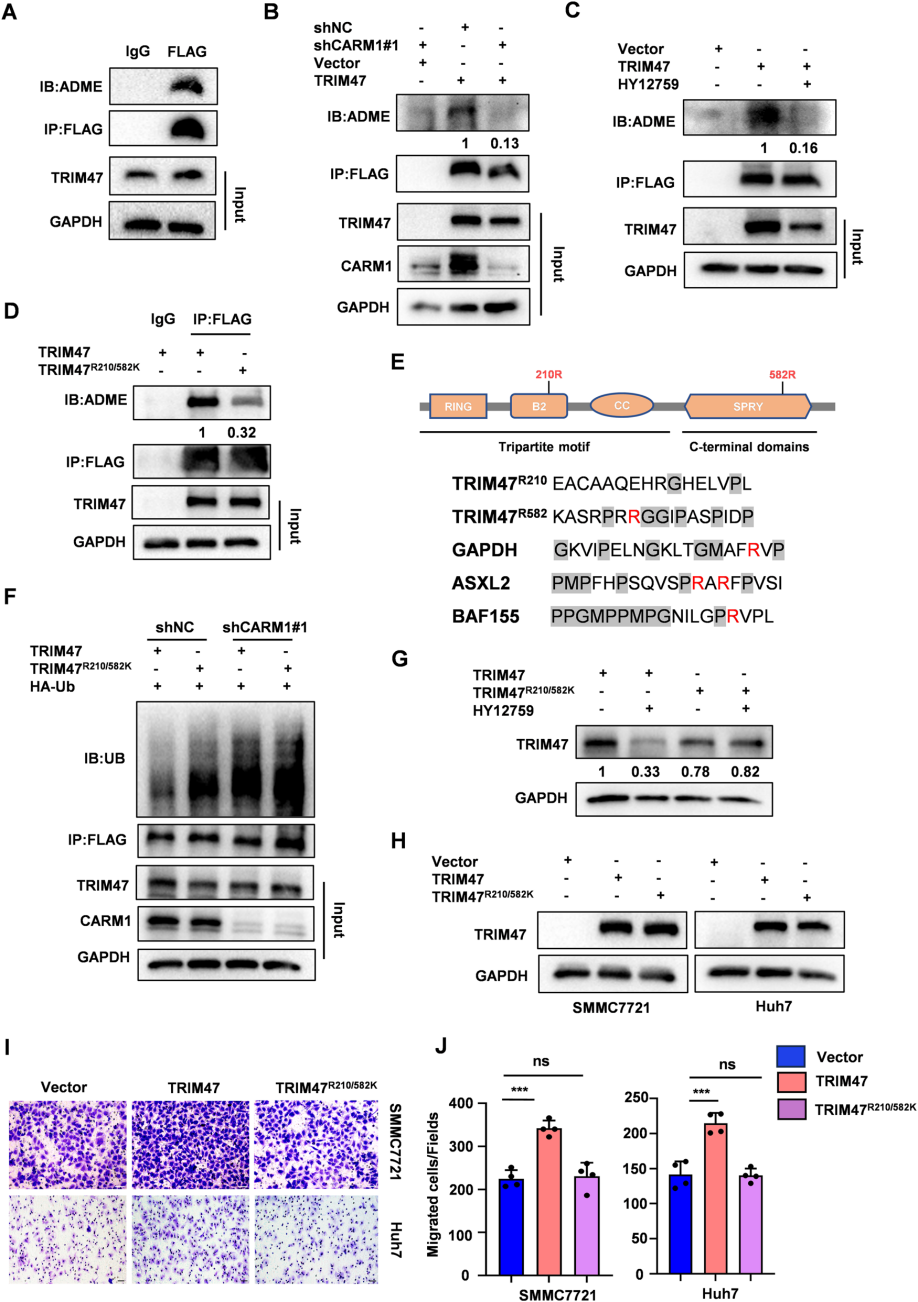
CARM1 di-methylates TRIM47 at its arginine 210 and arginine 582. **A** SMMC7721 cells were transfected with FLAG-TRIM47 for 48 h. Cell extracts were subjected to immunoprecipitated with anti-FLAG antibody or control IgG. Arginine methylation of immunopurified TRIM47 was detected by western blot. **B** CARM1 stably knockdown (shCARM1#1) or control (shNC) SMMC7721 cells were transfected with Vector or FLAG-TRIM47 for 48 h. Cell extracts were subjected to immunoprecipitated with anti-FLAG antibody. Arginine methylation of immunopurified TRIM47 was detected by western blot. **C** SMMC7721 cells were transiently transfected with Vector or FLAG-TRIM47 for 48 h, then treated with 10 μM HY12759 for another 8 h. Arginine methylation of immunopurified TRIM47 was detected by western blot. **D** SMMC7721 cells were transfected with FLAG-TRIM47 or FLAG-TRIM47^R210/582K^ for 48 h. Cell extracts were subjected to immunoprecipitated with anti-FLAG antibody. Arginine methylation of immunopurified TRIM47 was detected by western blot. **E** The schematic representation of TRIM47 constructs (Upper panel). The conserved glycine-arginine-methionine (PGM) rich substrate motif reported for the recognition of CARM1 (bottom panel). **F** CARM1 stably knockdown (shCARM1#1) or control (shNC) SMMC7721 cells were co-transfected with FLAG-TRIM47 or FLAG-TRIM47^R210/582K^ and HA-ubiquitin plasmids for 48 h. Cell extracts were immunoprecipitated with anti-FLAG antibody. The immunoprecipitates were detected with anti-ubiquitin and anti-FLAG antibodies. **G** SMMC7721 cells were transfected with FLAG-TRIM47 or TRIM47^R210/582K^ for 48 h and treated with HY12759 for another 8 h. Western blot analysis of TRIM47 protein levels. **H** SMMC7721 cells were transfected with FLAG-TRIM47 plasmid for 48 h and treated with HY12759 for another 8 h. Cell extracts were immunoprecipitated with anti-FLAG antibody. The immunoprecipitates were detected with anti-ubiquitin and anti-FLAG antibodies. **H** Western blot analysis of TRIM47 protein levels in SMMC7721 and Huh7 cells transiently transfected with Vector, FLAG-TRIM47 or FLAG-TRIM47^R210/582K^. **I**, **J** The migration assay of SMMC7721 and Huh7 cells transiently transfected with Vector, FLAG-TRIM47 or FLAG-TRIM47^R210/582K^. (**I**). The average number of cells per field were calculated (**J**). Scale bars, 50 μm. Data shown as mean ± SD. n = 3 samples per group, four fields per sample. ***, *P* < 0.001, ns no significance.

As a type I PRMTs, CARM1 catalyzes the formation of asymmetric di-methylarginine in its substrate proteins [9]. We found that TRIM47 was indeed arginine-methylated by using an anti-asymmetric di-methylarginine (α-ADME) antibody (Figs. 3A and S2A, B). CARM1 knockdown or HY-12759 treatment markedly decreased the methylation of TRIM47 (Fig. 3B, C). To define which arginine residues were methylated by CARM1, we performed mass spectrometric analysis of immunopurified TRIM47 protein from HCC cells and identified arginine 210 (R210) as a putative asymmetric di-methylation site (Fig. S3C). A previous proteomic study reported that arginine 582 (R582) might be another methylated arginine residue of TRIM47 [15]. To determine whether R210 and R582 are the major methylated site in TRIM47, we mutated these two sites into lysine (TRIM47^R210K^ or TRIM47^R582K^) that conferred resistance to arginine methylation. Compared with wild-type (WT) TRIM47, both TRIM47^R210K^ and TRIM47^R582K^ mutants showed a substantial, but incomplete reduction of asymmetric di-methylation levels (Fig. S3D). The methylation signal was also markedly inhibited for the R210 and R582 double mutant (TRIM47^R210/582K^) (Fig. 3D). These results indicate that both R210 and R582 are the major, if not the sole, methylation site of TRIM47. Interestingly, R582 is located in a conserved glycine-arginine-methionine (PGM) rich substrate motif reported for the recognition of CARM1, while R210 is not (Fig. 3E). Moreover, TRIM47^R210/582K^ mutant failed to interacting with CARM1 (Fig. S3E). We further tested whether the methylation of R210 and R582 contributed to TRIM47 stability and ubiquitination status. TRIM47^R210/582K^ mutant exhibited higher ubiquitination levels than that of WT TRIM47 (Fig. S3F). The ubiquitination level of WT TRIM47 was sharply enhanced by CARM1 knockdown, whereas TRIM47^R210/582K^ mutant showed little or no change (Fig. 3F). Consistently, HY-12759 significantly decreased WT TRIM47 protein levels, but not TRIM47 mutants (TRIM47^R210K^, TRIM47^R582K^ and TRIM47^R210/582K^) (Figs. 3G and S3G). We also assessed the effect of TRIM47 arginine mutants on the metastasis of HCC. TRIM47^R210K^ and TRIM47^R582K^ mutant-expressing cells were less metastatic compared to the WT TRIM47-expressing cells (Fig. S3H–J). Noticeably, TRIM47^R210/582K^ mutant absolutely abolished the pro-metastatic ability of TRIM47 in HCC (Figs. 3H–J and S3H–J). Together, these data indicated that the methylated modification by CARM1 stabilizes TRIM47 protein levels and promotes the metastasis of HCC.

### CARM1 suppresses HCC metastasis in a TRIM47-dependent manner

**Fig. 4:**
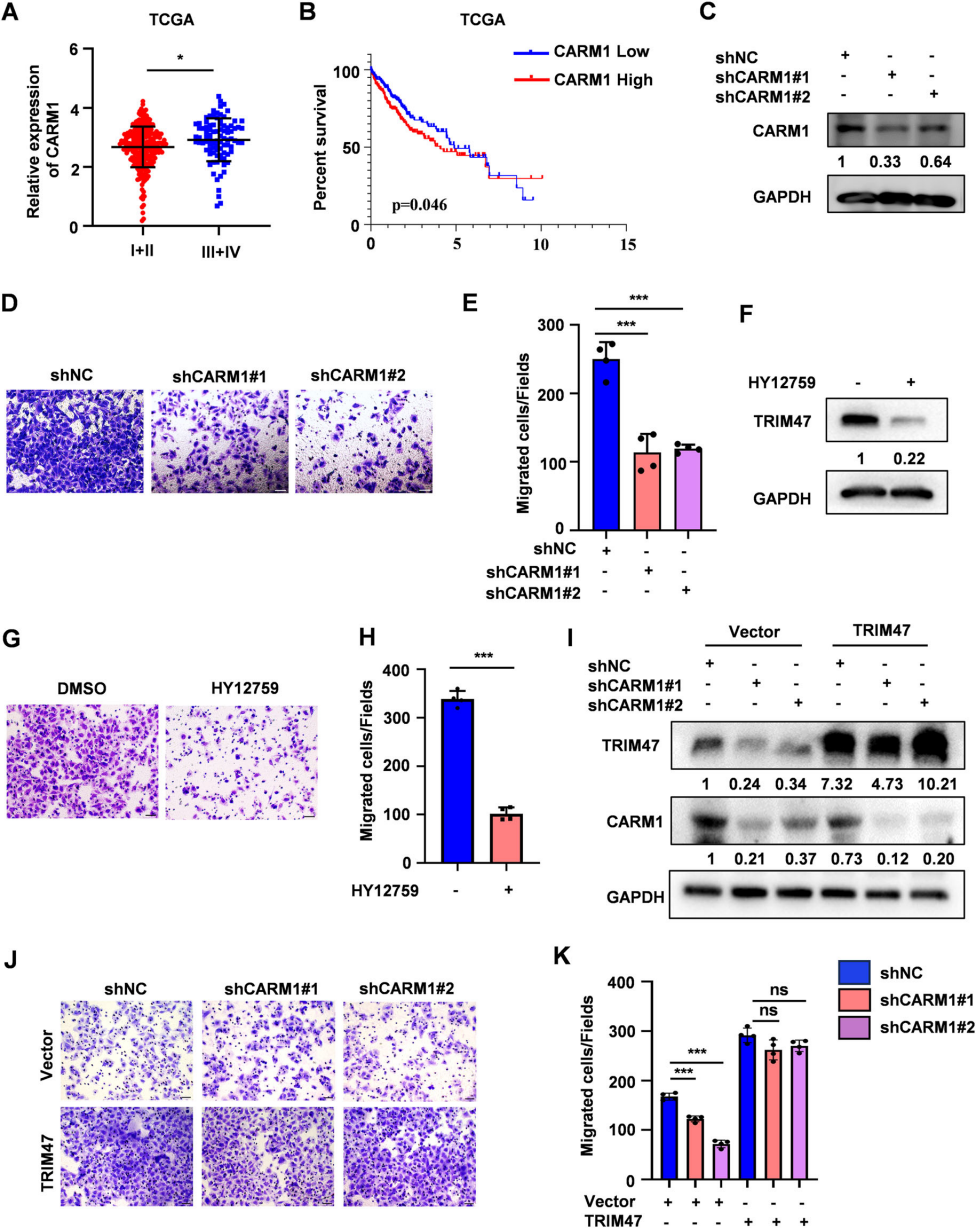
CARM1 promotes HCC cells migration depend on TRIM47*.* **A** Analysis of CARM1 mRNA levels in different tumor stages (stag III + IV vs stag I + II) of HCC patients from TCGA database. *P < 0.05. **B** Kaplan–Meier survival analysis of overall survival (OS) stratified by CARM1 expression in the HCC tissues from TCGA database. *P* = 0.046. **C** Western blot analysis of CARM1 protein levels in CARM1 stably knockdown (shCARM1#1 and shCARM1#2) or control (shNC) SMMC7721 cells. **D**, **E** The migration assay of SMMC7721 cells with CARM1 stably knockdown (**D**). The average number of cells per field were calculated (**E**). Scale bars, 50 μm. Data shown as mean ± SD. *n* = 3 samples per group, four fields per sample. ***P < 0.001. **F** Western blot analysis of TRIM47 protein levels in SMMC7721 cells treated with HY12795 (10 μM) for 8 h. **G**, **H** The migration assay of SMMC7721 cells treated with HY12795 (10 μM) for 8 h (**G**). The average number of cells per field were calculated (**H**). Scale bars, 50 μm. Data shown as mean ± SD. n = 3 samples per group, four fields per sample. ***P < 0.001. **I**–**K** CARM1 stable knockdown SMMC7721 cells were transfected with Vector or FLAG-TRIM47. Cells were harvested and subjected to western blot (**I**) and migration assay (**J**). The average number of cells per field were calculated (**K**). Scale bars, 50 μm. Data shown as mean ± SD. *n* = 3 samples per group, four fields per sample. ***P < 0.001; ns no significance.

CARM1 functions as an oncogene or a tumor suppressor depending on cancer types [16, 17]. However, the roles of CARM1 in dynamic HCC progression are controversial [12, 13]. We analyzed the HCC cohort in TCGA and found that HCC patients at the advanced stages expressed higher CARM1 than early staged patients (stage III + IV vs stage I + II) (Fig. 4A). Kaplan-Meier survival analysis revealed that high levels of CARM1 was associated with poor survival in HCC patients (Fig. 4B, Fig. S4A). Silencing of CARM1 or CARM1 inhibitor treatment suppressed the migration ability of SMMC7721 cells while CARM1 overexpression promoted cell migration, which were similar to the phenotypes observed for TRIM47 (Figs. 4C–H and S4B–D). Moreover, TRIM47 overexpression completely rescued the migration inhibition induced by CARM1 knockdown (Fig. 4I–K). Thus, we concluded that CARM1 promotes HCC metastasis in a TRIM47-dependent manner.

### TRIM47 methylation inhibits the ubiquitylation of TRIM47 by CUL4^CRBN^

**Fig. 5:**
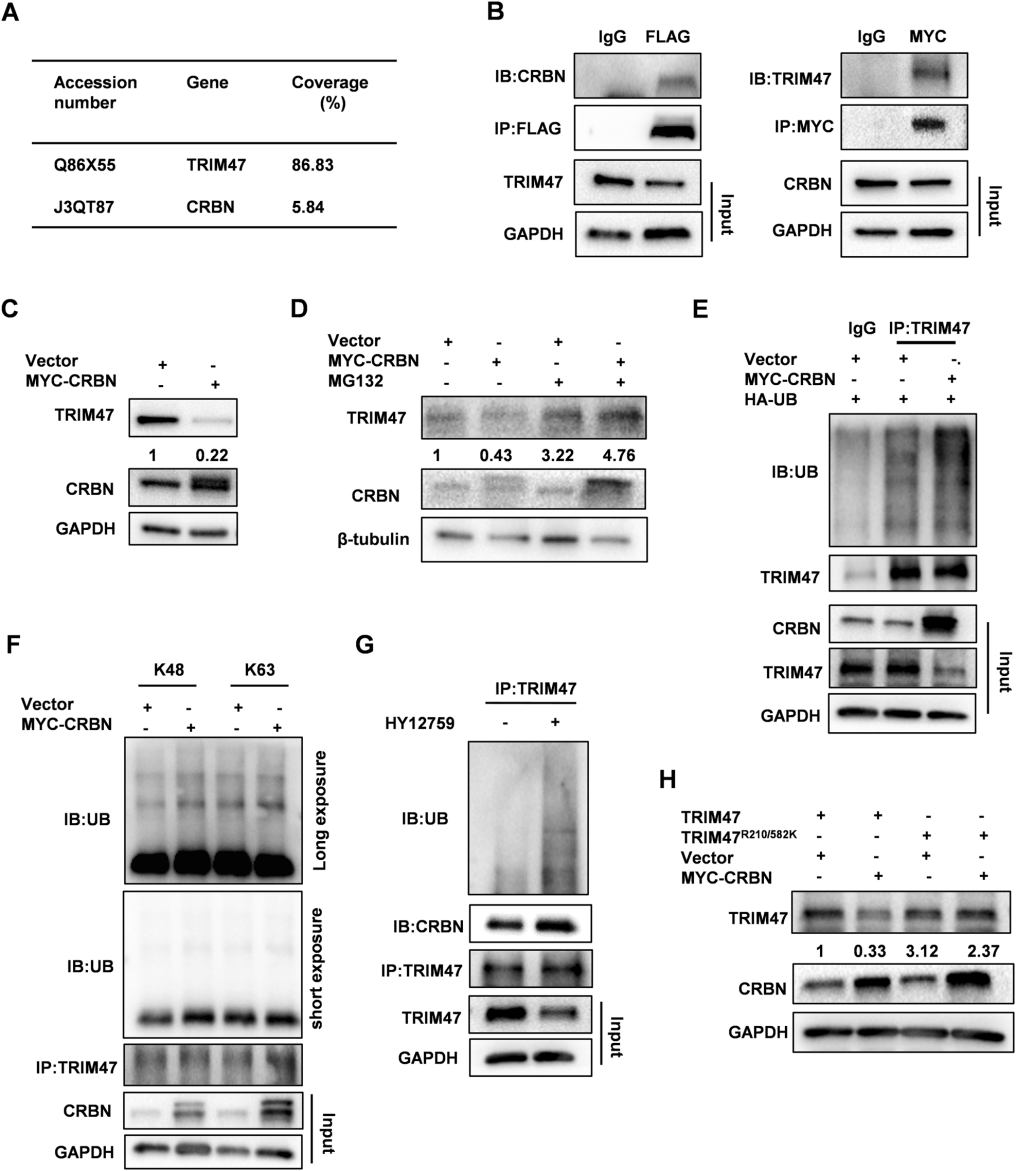
CRBN promotes the ubiquitination of TRIM47. **A** HEK293T cells were transfected with FLAG-TRIM47 for 48 h. Cell extracts were subjected to immunoprecipitated with anti-FLAG antibody or a control IgG. CRBN was identified via mass spectrometry. **B** HEK293T cells were co-transfected with FLAG-TRIM47 and MYC-CRBN for 48 h. Cell extracts were immunoprecipitated with anti-FLAG or anti-MYC antibodies. FLAG-TRIM47 and MYC-CRBN were detected by western blot. **C** Western blot analysis of TRIM47 protein levels in SMMC7721 cells transfected with MYC-CRBN for 48 h. **D** SMMC7721 cells were transfected with MYC-CRBN plasmid for 48 h and treated with MG132 (10 μM) for another 4 h. Western blot analysis of TRIM47 protein levels. **E** SMMC7721 cells were co-transfected with MYC-CRBN and HA-ubiquitin plasmids for 48 h. Cell extracts were immunoprecipitated with anti-TRIM47 antibody. The immunoprecipitates were detected with anti-ubiquitin and anti-TRIM47 antibodies. **F** Immunoprecipitation analysis of TRIM47 ubiquitination in SMMC7721 cells co-transfected with MYC-CRBN and HA-ubiquitin (K48) or HA-ubiquitin (K63). Cell extracts were immunoprecipitated with anti-TRIM47 antibody. The immunoprecipitates were detected with anti-HA and anti-TRIM47 antibodies. **G** SMMC7721 cells were treated with HY12759 for 8 h. Total cell extracts were immunoprecipitated with anti- TRIM47 antibody. The immunoprecipitates were detected with anti-ubiquitin, anti-TRIM47 and anti-CRBN antibodies. **H** Western blot analysis of TRIM47 protein levels in TRIM47 stably knockdown SMMC7721 cells co-transfected with FLAG-TRIM47 or FLAG-TRIM47^R210/582K^ and MYC-CRBN plasmids for 48 h.

Previous results suggested that CARM1 stabilized TRIM47 protein levels by inhibiting its ubiquitylation and degradation (Fig. 2F–H). We intended to identify the E3 ubiquitin ligase that mediated the degradation of TRIM47. The above immunoprecipitation-coupled mass spectrometry screen conducted by us found that CRBN, a substrate adapter for the CRL4 E3 ubiquitin ligase, was a potential TRIM47 interacting protein (Fig. 5A). Co-IP experiments showed that TRIM47 indeed interacted with CRBN (Fig. 5B). TRIM47 protein level was decreased by CRBN overexpression, which could be blocked by MG132 (Fig. 5C, D). Consistently, CRBN overexpression promoted the ubiquitination of TRIM47 (Fig. 5E). Since CRL4 E3 ubiquitin ligases employed CRBN as an adaptor to recognize substrate protein [18], we detected one of the CUL4 paralogues, CUL4A for the ubiquitination and protein stability of TRIM47. CUL4A overexpression also significantly enhanced the ubiquitination and degradation of TRIM47 (Fig. S5A, B). Using the ubiquitin mutants with the same lysine at position 48 or 63 and the remaining lysine replaced by arginine (K48 or K63), we found that CRBN enhanced K48-linked ubiquitination of TRIM47 (Fig. 5F and S4C). Moreover, HY15729 strengthened the interaction between CRBN and TRIM47 in SMMC7721 cells, leading the enhancement of TRIM47 ubiquitination. We further investigated whether CARM1-mediated methylation affected TRIM47 ubiquitylation regulated by CUL4^CRBN^. CRBN overexpression only decreased the protein level of WT TRIM47, whereas TRIM47^R210/582A^ remained unchanged (Fig. 5H). This novel observation reveals a crosstalk between methylation and ubiquitylation in the orchestration of TRIM47 protein stability.

### TRIM47 promotes HCC metastasis by protecting SNAI1 from proteasome-mediated degradation

**Fig. 6:**
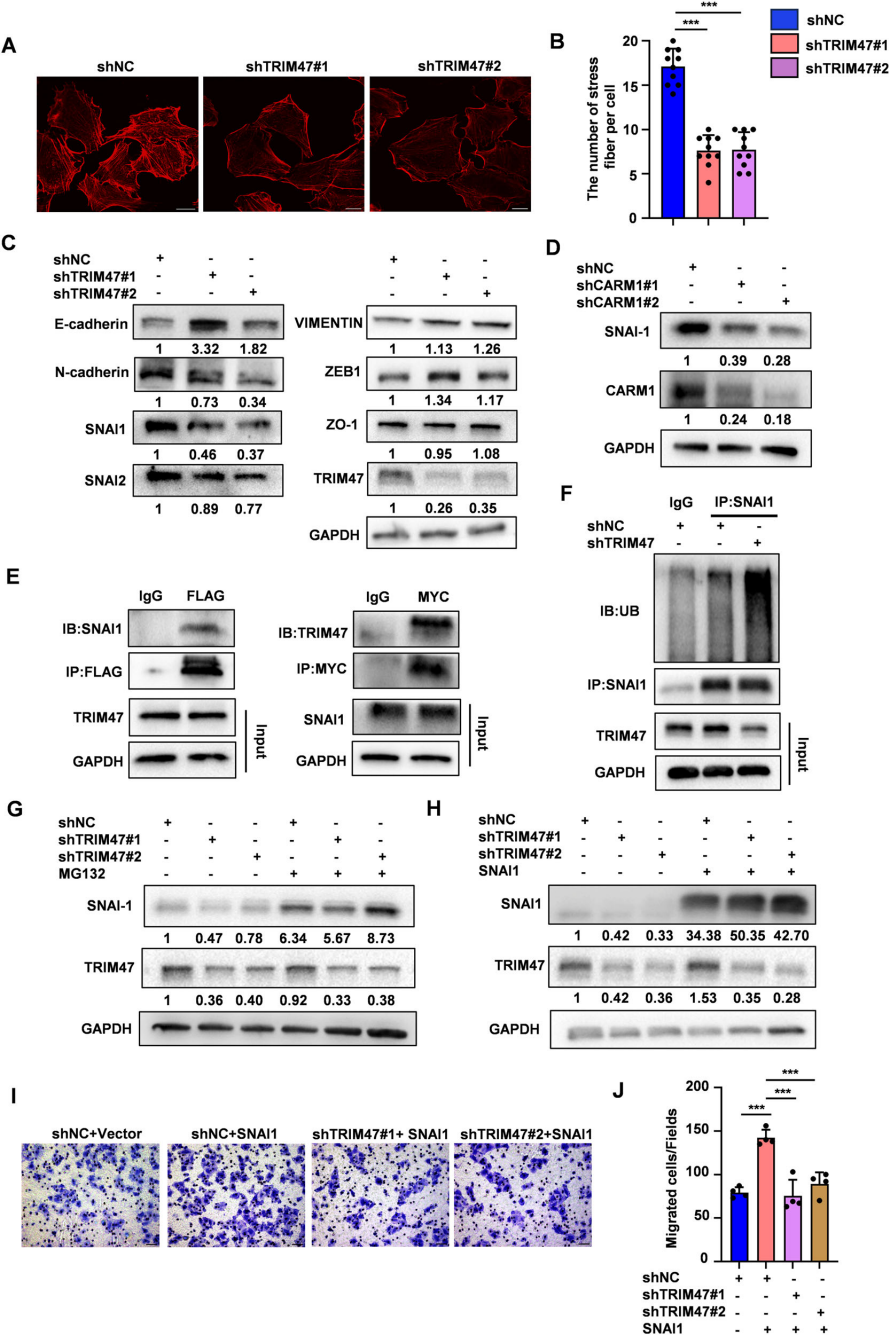
TRIM47 promotes HCC metastasis via SNAI1. **A**, **B** Representative images of F-actin (red) staining in control (shNC) and TRIM47 stably knockdown (shTRIM47#1 and shTRIM47#2) SMMC7721 cells (**A**). Quantification of stress fibers per cell (**B**). Scale bars, 200 μm. Data are shown as mean ± SD. n = 10 cells per condition, ***P < 0.001. **C** Western blot analysis of EMT related proteins, including CDH1, CDH2, SNAI1, SNAI2, VIMENTIN, ZEB-1 and ZO-1 in TRIM47 stably knockdown SMMC7721 cells. **D** Western blot analysis of SNAI1 protein levels in CARM1 stably knockdown SMMC7721 cells. **E** HEK293T cells were co-transfected with FLAG-TRIM47 and MYC-SNAI1 for 48 h. Cell extracts were immunoprecipitated with anti-FLAG or anti-MYC antibodies. FLAG-TRIM47 and MYC-SNAI1 were detected by western blot. **F** Cell extracts from TRIM47 stably knockdown (shTRIM47#1) or control (shNC) SMMC7721 cells were immunoprecipitated with anti-SNAI1 antibody. The immunoprecipitates were detected by with anti-ubiquitin and anti-SNAI1 antibodies. **G** TRIM47 stably knockdown (shTRIM47#1 and shTRIM47#2) or control (shNC) SMMC7721 cells were treated with DMSO or 10 μM MG132 for 6 h. Western blot analysis of SNAI1 protein levels. **H**–**J** TRIM47 stably knockdown SMMC7721 cells were transfected with Vector or MYC-SNAI1. Cells were harvested and subjected to Western blot (**H**) and migration assay (**I**). The average number of cells per field were calculated (**J**). Scale bars, 50μm. Data shown as mean ± SD. *n* = 3 samples per group, four fields per sample. ***P < 0.001.

We next explored the potential mechanisms by which CARM1-TRIM47 axis suppressed HCC metastasis. TRIM47 knockdown significantly decreased the number of F-actin stress fibers, which is the hallmark of the cell migration (Fig. 6A, B). We examined several pathways and molecular that influence cell metastasis, including canonical WNT/β-catenin signaling [19], RhoA/ROCK1 signaling [20], RAC1 pathway [21] and EMT inducing transcription factors [22]. Interestingly, only SNAI1 protein levels were significantly decreased in TRIM47 silenced cells (Figs. 6C and S6A–C). Knockdown of CARM1 also inhibited SNAI1 protein expression (Fig. 6D). Interestingly, Co-IP experiments showed that SNAI1 is a binding partner of TRIM47 (Fig. 6E). Of note, Treatment with MG132, not with CQ, could rescue the reduced SNAI1 protein levels by TRIM47 knockdown (Figs. 6G and S6D), suggesting that TRIM47 knockdown promotes SNAI1 degradation via the ubiquitin-proteasome pathway. To validate this, we performed protein ubiquitination assays. The ubiquitination of SNAI1 was enhanced by TRIM47 knockdown (Fig. 6F). SNAI1 overexpression extensively reversed the suppressive effect of TRIM47 depletion on migration of SMMC7721 cells (Fig. 6H–G). Collectively, these results indicate that TRIM47 promotes HCC cells migration by protecting SNAI1 from proteasome-mediated degradation.

## Discussion and conclusion

TRIM47 is over-expressed and acts as an oncogene in multiple types of tumors, such as prostate cancer [5], colorectal cancer [4] and breast cancer [23]. TRIM47 facilitates breast cancer proliferation and endocrine therapy resistance by activating NF-κB signaling [24]. Up-regulated TRIM47 is associated with poor outcomes and promotes the proliferation and metastasis of CRC [4]. However, the biological functions of TRIM47 and the possible underlying mechanisms in HCC have not been elucidated. In this study, we showed that the TRIM47 was up-regulated in advanced HCC and associated with poor survival of HCC patients. TRIM47 was able to promote HCC metastasis in vitro and in vivo. Therefore, TRIM47 acted as a pro-metastatic factor in HCC, which was consistent with previous study demonstrated that a TRIM family gene-based signature including TRIM47 and another 5 TRIM genes could be used to predict poor prognosis of HCC with high accuracy [7].

Although TRIM47’s role in a variety of tumors has been extensively reported, little is known about its regulatory mechanisms in cancer. A study reported that LMO7 promoted the lysine 48 (K48)-linked ubiquitination and degradation of TRIM47 to alleviate hepatic steatosis, inflammation, and fibrosis [25]. Our study identified TRIM47 as a previously uncharacterized substrate of CARM1 and CARM1 methylated TRIM47 at its arginine 210 and arginine 582. The canonical CARM1 methylation motif is PMG rich motifs [15]. The TRIM47^R582^ methylation residue fit the consensus motif while TRIM47^R210^ do not. The crosstalk between methylation and other modifications regulates protein stability, activity, and intracellular location. PRMT5 methylated Mxi1 and facilitated its ubiquitin-mediated degradation in lung cancer [26]. The methylation of FOXO1 at its residues Arg 248 and Arg 250 by PRMT1 blocks AKT dependent phosphorylation of FOXO1, thereby increasing the stability and transcriptional activity of FOXO1 [27]. Our study found that the methylation of TRIM47^R210/582^ could be used to mark it for degradation by the CUL4^CRBN^ E3 ubiquitin ligase complex. Interestingly, besides stabilizing the protein levels of TRIM47, TRIM47 ^R210/582^ methylation directly affect its ability to promote HCC cells migration.

CARM1 methylates histone and non-histone proteins to function as an oncogene or tumor suppressor in different types of malignant tumors, suggesting that the function of CARM1 in cancer is context-dependent [11, 28, 29]. Compared with defined roles in other cancers, the function of CARM1 in HCC is controversial, as both pro-tumorigenic and onco-suppressor functions were reported [12, 13, 30]. Here, we found that CARM1 had a similar pro-metastasis phenotype with TRIM47 in HCC. High expression of CARM1 was correlated with poor prognosis in HCC patients and CARM1 was able to promote HCC metastasis in vitro and in vivo. Moreover, the promotion of HCC metastasis mediated by CARM1 was largely dependent on TRIM47. Our data strongly supports CARM1 as an oncogene in HCC. However, large-scale cohort studies and detailed immunohistochemical analysis of TRIM47 and CARM1 in HCC clinical samples are still needed for future investigation, which may facilitate understanding their roles and clinical correlation in HCC progression.

As an E3 ubiquitin ligase, TRIM47 usually promoted the ubiquitination and degradation of its binding proteins, such as SMAD4 and FOXO1 [4, 31]. However, we observed that TRIM47 interacted with SNAI1 and protected it from ubiquitin-mediated degradation. SNAI1 is an important transcription factor of EMT, which suppresses the transcription of E-cadherin by combining with the E-box sequence of E-cadherin’s promoter [32]. EMT is characterized by reduced cell-cell adhesion, lossing of cell polarity and the acquisition of mesenchymal features, which is considered to be a prerequisite for tumor metastasis [33, 34]. A study reported that TRIM47 knockdown inhibited EMT through the inactivation of Wnt/β-catenin pathway in glioma [35]. To our surprise, TRIM47 knockdown did not affect Wnt/β-catenin pathway in HCC cells. The ubiquitination of SNAI1 was regulated by several E3 ubiquitin ligase, including FBXO11 [36], GSK3β [37], and FBXL14 [38]. The interaction between TRIM47 and SNAI1 may prevent the ubiquitylation and degradation of SNAI1 by other E3 ubiquitin ligases.

**Fig. 7:**
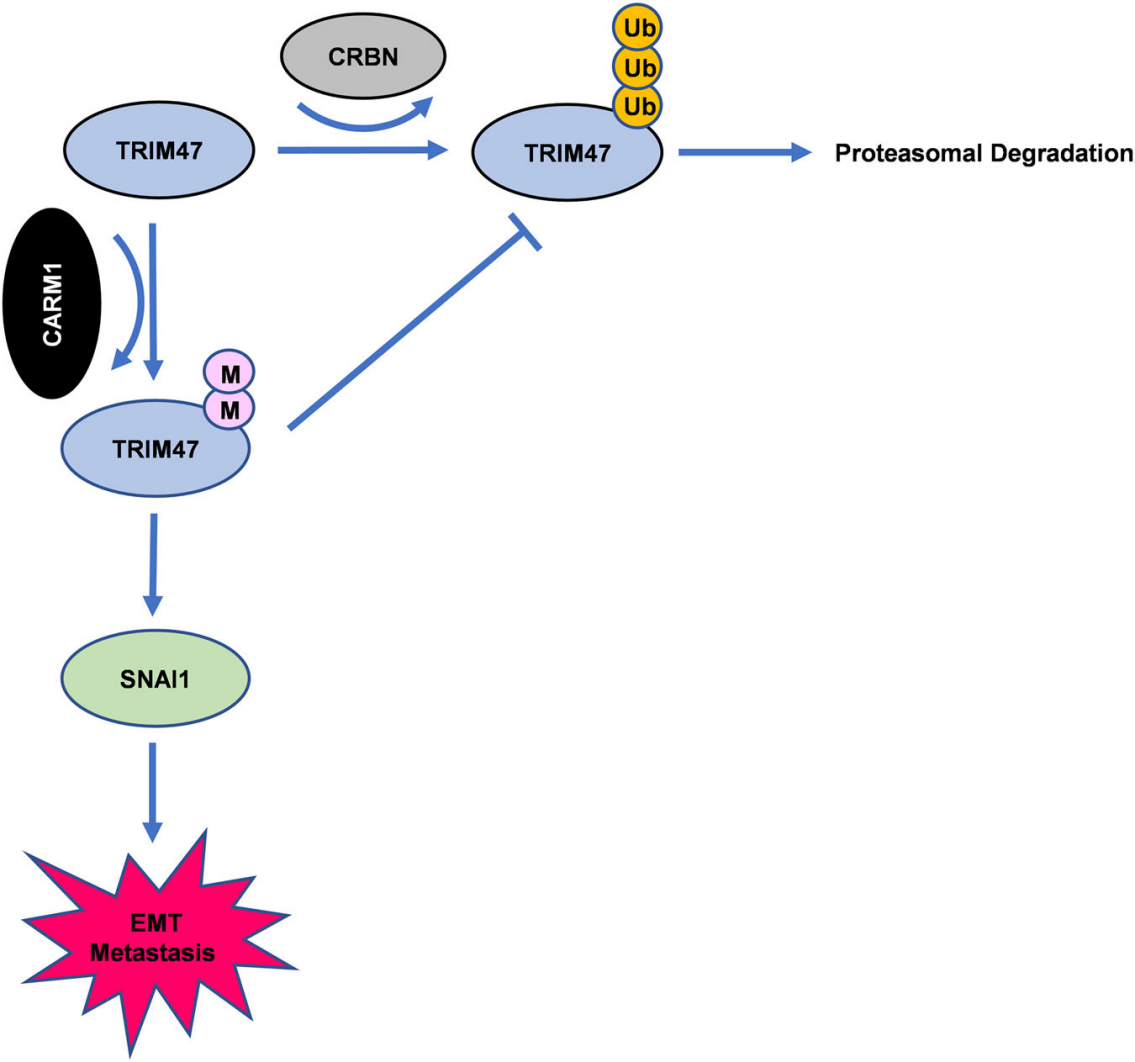
Proposed model showing the regulation of TRIM47 by CARM1.

In conclusion, we display for the first time that CARM1-CUL4^CRBN^-TRIM47-SNAI1 cascade is involved in HCC metastasis. Our data are consistent with a model in which TRIM47 methylation by CARM1 inhibits CUL4^CRBN^-mediated ubiquitylation of TRIM47. Accumulated TRIM47 thus interacts with SANI1 and protecting it from proteasomal degradation (Fig. 7). Our findings underscore the roles of TRIM47 and CARM1 in HCC metastasis, unveils a novel mechanism that crosstalk between arginine methylation and ubiquitylation orchestrates TRIM47-mediated HCC metastasis. The newly defined CARM1-CUL4^CRBN^-TRIM47-SNAI1 regulatory axis may open new avenues for metastatic HCC therapy.

## Materials and methods

### Antibodies and reagents

Antibodies used in this study were as follows: anti-TRIM47 (Proteintech, 26885-1-AP), CARM1 (Proteintech, 55246-1-AP), anti-GAPDH (Proteintech, 10494-1-AP), anti-Ubiquitin (Proteintech, 10201-2-AP), anti-Myc (Abclonal, AE010), anti-FLAG (Abclonal, AE005), Asymmetric di-methyl arginine antibody (Cell Signaling Technology, 13522), anti-E-cadherin (Proteintech,20874-1-AP), anti-N-cadherin (Proteintech, 22018-1-AP), anti-RHOA (Proteintech, 10749-1-AP), anti-Rac1 (Proteintech, 24072-1-AP), anti-AXIN2 (Proteintech, 20540-1-AP), anti-β-Catenin (Proteintech, 51067-2-AP), anti-GST (Proteintech,10000-0-AP), anti-CRBN (Proteintech, 28494-1-AP), anti-SNAI1 (Proteintech, 13099-1-AP), anti-SNAI2 (Cell Signaling Technology, 9585), anti-Vimentin (Cell Signaling Technology, 5741), anti-ZEB1 (Cell Signaling Technology, 3396), anti-ZO-1(Cell Signaling Technology, 5406). Other reagents used in this study were: Cycloheximide (MedchemExpress, HY-12320), CARM1-IN-1 (MedchemExpress, HY-12759), MG132 (MedchemExpress, HY-13259), Chloroquine (Sigma-Aldrich, C6628-25G).

### Cell culture and transfection

HEK293T and human HCC cell lines HepG2, Huh7 were obtained from American Type Culture Collection (ATCC). SMMC7721 cell line was purchased from Warner Bio with STR. All cells used in this study were cultured in RPMI 1640 medium (Gibco, 31800022) and DMEM medium (Gibco, 12800017) containing 10% fetal bovine serum (VISTECH, SE100-011). Plasmids were transiently transfected into cells by using Neofect^TM^DNA reagent (Neofect, TF20121201) according to the manufacturer’s instruction.

### Plasmids construction

Human TRIM47 cDNA was cloned into the p3×FLAG-CMV-10 Vector (sigma aldrich) to generate p3×FLAG-CMV-TRIM47. Human CARM1, CRBN and SNAI1 cDNA was cloned into pcDNA.3.1 Vector (Invitrogen). The following mutational TRIM47 plasmids were constructed based on p3×FLAG-CMV-TRIM47 plasmid: p3×FLAG-CMV-TRIM47^R210K^, p3×FLAG-CMV-TRIM47^R582K^, p3×FLAG-CMV-TRIM47^R210/582K^. TRIM47 and TRIM47^R210/582K^ stably overexpression cell lines were constructed using the pLenti-3×Flag-CMV plasmid. Oligonucleotides specific shRNA against TRIM47 or CARM1 were synthesized and cloned into pLKO.1 Vector (Addgene Plasmid, #10878). All plasmids used in our study were confirmed by DNA sequencing. The shRNA sequences were listed in Table S1.

### Lentivirus production and transduction

Indicated lentiviral plasmids were transfected into HEK293T cells. After 48 h post transfection, the supernatant containing lentiviral particles was harvested. HCC cells were infected with the lentivirus in the presence of polybrene (8 μg/mL) for 24 h and then selected by puromycin (0.5 μg/mL) for one week. The overexpression or knockdown efficiency was detected by western blot.

### Cell migration, invasion and wound healing assay

The cell migration and invasion assay were performed by using 8-μm Boyden chambers (Corning Inc, 3422). For migration assay, 1 × 10^5^ SMMC7721 cells or Huh7 cells were seeded in the upper chamber with serum-free RPMI 1640 medium. The lower chamber was added with RPMI 1640 medium containing 10% FBS. After 12 h for SMMC7721 or 6 h for Huh7, the migrated cells were fixed with 4% paraformaldehyde, stained with crystal violet and counted.

For invasion assay, the upper chamber was pre-coated with matrigel matrix (BD Science, 356234). 2 × 10^5^ SMMC7721 cells were seeded in the upper chamber with serum-free RPMI 1640 medium. The lower chamber was added with RPMI 1640 medium containing 10% FBS. After 72 h, the invaded cells were fixed with 4% paraformaldehyde, stained with crystal violet and counted.

For wound healing assay, indicated cells were seeded in 6-well plate and grown to 90% confluence. A linear scratch was made by a sterile 200 μl tip. Cells were cultured in RPMI 1640 medium containing 1% FBS to close the wound for 48 h. The scratch area was analyzed by Image J software.

### Animal experiments

1 × 10^6^ indicated SMMC7721 cells (shNC, shTRIM47#1 and shTRIM47#2) were injected into Balb/c nude mice (male, 6 weeks) through tail vein. After 8 weeks, the mice were sacrificed and pulmonary metastatic nodules were counted. The animal studies were approved by the Animal Ethics Committee of Wuhan University of Science and Technology. The mice used in our study were housed under specific pathogen-free (SPF) conditions.

### ***RNA Extraction and*** Semi-Quantitative RT-PCR

RNA was extracted by RNA extraction kit (Abclonal, RK30120) and cDNA was synthesized using a Transcription Reagent Kit (Abclonal, RK20429) according to the manufacturer’s instruction. PCR analysis of mRNA expression was conducted using a 2 × Taq Plus Master Mix (Vazyme, P211-01). The PCR products were separated by agarose gel electrophoresis.

### Immunofluorescence and F-actin staining

Cells were fixed with 4% paraformaldehyde and permeabilized with 0.2% Triton X-100 (Sigma-Aldrich). For immunofluorescence, cells were then blocked with 10% FBS, incubated with the indicated primary antibodies and secondary antibodies. For F-actin staining, cells were incubated with Rhodamine-conjugated Phalloidins for 1 h. nuclei was stain with DAPI.

### Western blot

Cells were harvested and lysed with RIPA buffer (Beyotime Biotechnology, P0013C) containing protease inhibitors. The protein concentration was detected with BCA kit (Thermo Scientific, A55864). The cell lysate was separated by SDS-PAGE and then transferred onto Immobilon-P (PVDF) membranes (Merck Millipore, ISEQ00010). After blocking with 5% skimmed milk, membranes were incubated with indicated antibodies. The protein bands were visualized by chemiluminescence system.

### Co-immunoprecipitation assay (Co-IP)

Cells were harvest and lysed with RIPA buffer (Beyotime Biotechnology, P0013D) containing protease inhibitors. The cell lysates were incubated with indicated antibodies overnight at 4 °C. Then protein A/G magnetic beads (Biolinkedin) were added and incubated for another 2 h. The protein-bound beads were washed with washing buffer for 3 times, boiled with protein loading buffer and analyzed by western blot.

### Liquid chromatograph–mass spectrometry (LC–MS) analysis

HEK293T cells were transfected with FLAG-TRIM47 for 48 h. Cell extracts were subjected to immunoprecipitated with anti-FLAG antibody or a control IgG. the immunoprecipitated proteins were eluted and then subjected to liquid chromatograph-mass spectrometry (LC–MS) analysis.

### GST-PAK1^PBD^ pull down assay and GTP-RhoA activation assay

GST-PAK1^PBD^ protein was expressed in *E. coli* strain BL21 and purified. Equal amounts of GST-PAK1^PBD^ protein were incubated with glutathione Sepharose 4B beads (GE Healthcare) and indicated cell lysates. Then the beads were washed with washing buffer for 5 times, boiled with protein loading buffer and analyzed by western blot.

The GTP-RhoA activation assay were performed with the Rho Activation Assay Biochem Kit (Cytoskeleton) according to the manufacture’s instruction.

### Statistical analysis

Kaplan–Meier method was performed to analyze survival rate and the survival differences was calculated with log-rank test. Unpaired Student’s t-tests or one-way ANOVA were used to determined other comparisons according to the number of groups. Date was presented as the mean ± standard deviation (SD). Statistical significance was set at *P* < 0.05. *P < 0.05, **P < 0.01, ***P < 0.001, ns, no significance. All analyses were performed with GraphPad Prism software.

## Supplementary information

Supplementary Materials

Original image for western blot

## Data Availability

The data are available in the article and obtained from the corresponding author upon reasonable request.

## References

[1] Di Rienzo M, Romagnoli A, Antonioli M, Piacentini M, Fimia GM. TRIM proteins in autophagy: selective sensors in cell damage and innate immune responses. Cell Death Differ. 2020;27:887–902. 31969691 10.1038/s41418-020-0495-2 PMC7206068

[2] Huang N, Sun X, Li P, Liu X, Zhang X, Chen Q, et al. TRIM family contribute to tumorigenesis, cancer development, and drug resistance. Exp Hematol Oncol. 2022;11:75. 36261847 10.1186/s40164-022-00322-w PMC9583506

[3] Han Y, Tian H, Chen P, Lin Q. TRIM47 overexpression is a poor prognostic factor and contributes to carcinogenesis in non-small cell lung carcinoma. Oncotarget. 2017;8:22730–40. 28186994 10.18632/oncotarget.15188 PMC5410258

[4] Liang Q, Tang C, Tang M, Zhang Q, Gao Y, Ge Z. TRIM47 is up-regulated in colorectal cancer, promoting ubiquitination and degradation of SMAD4. J Exp Clin Cancer Res. 2019;38:159. 30979374 10.1186/s13046-019-1143-x PMC6461818

[5] Fujimura T, Inoue S, Urano T, Takayama K, Yamada Y, Ikeda K, et al. Increased expression of tripartite motif (TRIM) 47 is a negative prognostic predictor in human prostate cancer. Clin Genitourin Cancer. 2016;14:298–303. 26873435 10.1016/j.clgc.2016.01.011

[6] Ducreux M, Abou-Alfa GK, Bekaii-Saab T, Berlin J, Cervantes A, de Baere T, et al. The management of hepatocellular carcinoma. Current expert opinion and recommendations derived from the 24th ESMO/World Congress on Gastrointestinal Cancer, Barcelona, 2022. ESMO Open. 2023;8:101567. 37263081 10.1016/j.esmoop.2023.101567 PMC10245111

[7] Dai W, Wang J, Wang Z, Xiao Y, Li J, Hong L, et al. Comprehensive analysis of the prognostic values of the TRIM family in hepatocellular carcinoma. Front Oncol. 2021;11:767644. 35004288 10.3389/fonc.2021.767644 PMC8733586

[8] Blanc RS, Richard S. Arginine methylation: the coming of age. Mol Cell. 2017;65:8–24. 28061334 10.1016/j.molcel.2016.11.003

[9] Suresh S, Huard S, Dubois T. CARM1/PRMT4: making its mark beyond its function as a transcriptional coactivator. Trends Cell Biol. 2021;31:402–17. 33485722 10.1016/j.tcb.2020.12.010

[10] Wang L, Zhao Z, Meyer MB, Saha S, Yu M, Guo A, et al. CARM1 methylates chromatin remodeling factor BAF155 to enhance tumor progression and metastasis. Cancer Cell. 2014;25:21–36. 24434208 10.1016/j.ccr.2013.12.007 PMC4004525

[11] Wang YP, Zhou W, Wang J, Huang X, Zuo Y, Wang TS, et al. Arginine methylation of MDH1 by CARM1 inhibits glutamine metabolism and suppresses pancreatic cancer. Mol Cell. 2016;64:673–87. 27840030 10.1016/j.molcel.2016.09.028

[12] Zhong XY, Yuan XM, Xu YY, Yin M, Yan WW, Zou SW, et al. CARM1 methylates GAPDH to regulate glucose metabolism and is suppressed in liver cancer. Cell Rep. 2018;24:3207–23. 30232003 10.1016/j.celrep.2018.08.066

[13] Du P, Luo K, Li G, Zhu J, Xiao Q, Li Y, et al. PRMT4 promotes hepatocellular carcinoma progression by activating AKT/mTOR signaling and indicates poor prognosis. Int J Med Sci. 2021;18:3588–98. 34522186 10.7150/ijms.62467 PMC8436100

[14] Chen JX, Xu D, Cao JW, Zuo L, Han ZT, Tian YJ, et al. TRIM47 promotes malignant progression of renal cell carcinoma by degrading P53 through ubiquitination. Cancer Cell Int. 2021;21:129. 33622324 10.1186/s12935-021-01831-0 PMC7903798

[15] Shishkova E, Zeng H, Liu F, Kwiecien NW, Hebert AS, Coon JJ, et al. Global mapping of CARM1 substrates defines enzyme specificity and substrate recognition. Nat Commun. 2017;8:15571. 28537268 10.1038/ncomms15571 PMC5458078

[16] Zhao Z, Rendleman EJ, Szczepanski AP, Morgan MA, Wang L, Shilatifard A. CARM1-mediated methylation of ASXL2 impairs tumor-suppressive function of MLL3/COMPASS. Sci Adv. 2022;8:eadd3339. 36197977 10.1126/sciadv.add3339 PMC9534506

[17] Cui J, Hu J, Ye Z, Fan Y, Li Y, Wang G, et al. TRIM28 protects CARM1 from proteasome-mediated degradation to prevent colorectal cancer metastasis. Sci Bull. 2019;64:986–97. 10.1016/j.scib.2019.05.024 36659810

[18] Cheng J, Guo J, North BJ, Tao K, Zhou P, Wei W. The emerging role for Cullin 4 family of E3 ligases in tumorigenesis. Biochim Biophys Acta Rev Cancer. 2019;1871:138–59. 30602127 10.1016/j.bbcan.2018.11.007 PMC7179951

[19] Gajos-Michniewicz A, Czyz M. WNT/beta-catenin signaling in hepatocellular carcinoma: The aberrant activation, pathogenic roles, and therapeutic opportunities. Genes Dis. 2024;11:727–46. 37692481 10.1016/j.gendis.2023.02.050 PMC10491942

[20] Zeng Y, Cao Y, Liu L, Zhao J, Zhang T, Xiao L, et al. SEPT9_i1 regulates human breast cancer cell motility through cytoskeletal and RhoA/FAK signaling pathway regulation. Cell Death Dis. 2019;10:720. 31558699 10.1038/s41419-019-1947-9 PMC6763430

[21] Liu S, Yu M, He Y, Xiao L, Wang F, Song C, et al. Melittin prevents liver cancer cell metastasis through inhibition of the Rac1-dependent pathway. Hepatology. 2008;47:1964–73. 18506888 10.1002/hep.22240

[22] Giannelli G, Koudelkova P, Dituri F, Mikulits W. Role of epithelial to mesenchymal transition in hepatocellular carcinoma. J Hepatol. 2016;65:798–808. 27212245 10.1016/j.jhep.2016.05.007

[23] Liu F, Xie B, Ye R, Xie Y, Zhong B, Zhu J, et al. Overexpression of tripartite motif-containing 47 (TRIM47) confers sensitivity to PARP inhibition via ubiquitylation of BRCA1 in triple negative breast cancer cells. Oncogenesis. 2023;12:13. 36906594 10.1038/s41389-023-00453-7 PMC10008536

[24] Azuma K, Ikeda K, Suzuki T, Aogi K, Horie-Inoue K, Inoue S. TRIM47 activates NF-kappaB signaling via PKC-epsilon/PKD3 stabilization and contributes to endocrine therapy resistance in breast cancer. Proc Natl Acad Sci USA. 2021;118:e2100784118. 34433666 10.1073/pnas.2100784118 PMC8536392

[25] Wu T, Chen X, Xu K, Dai C, Li X, Zhang YW, et al. LIM domain only 7 negatively controls nonalcoholic steatohepatitis in the setting of hyperlipidemia. Hepatology. 2024;79:149–66. 37676481 10.1097/HEP.0000000000000585 PMC10718224

[26] Yang X, Zeng Z, Jie X, Wang Y, Han J, Zheng Z, et al. Arginine methyltransferase PRMT5 methylates and destabilizes Mxi1 to confer radioresistance in non-small cell lung cancer. Cancer Lett. 2022;532:215594. 35149174 10.1016/j.canlet.2022.215594

[27] Yamagata K, Daitoku H, Takahashi Y, Namiki K, Hisatake K, Kako K, et al. Arginine methylation of FOXO transcription factors inhibits their phosphorylation by Akt. Mol Cell. 2008;32:221–31. 18951090 10.1016/j.molcel.2008.09.013

[28] Gao G, Hausmann S, Flores NM, Benitez AM, Shen J, Yang X, et al. The NFIB/CARM1 partnership is a driver in preclinical models of small cell lung cancer. Nat Commun. 2023;14:363. 36690626 10.1038/s41467-023-35864-y PMC9870865

[29] Liu F, Ma F, Wang Y, Hao L, Zeng H, Jia C, et al. PKM2 methylation by CARM1 activates aerobic glycolysis to promote tumorigenesis. Nat Cell Biol. 2017;19:1358–70. 29058718 10.1038/ncb3630 PMC5683091

[30] Osada S, Suzuki S, Yoshimi C, Matsumoto M, Shirai T, Takahashi S, et al. Elevated expression of coactivator-associated arginine methyltransferase 1 is associated with early hepatocarcinogenesis. Oncol Rep. 2013;30:1669–74. 23912631 10.3892/or.2013.2651

[31] Wei H, Ding C, Zhuang H, Hu W. TRIM47 promotes the development of glioma by ubiquitination and degradation of FOXO1. Onco Targets Ther. 2020;13:13401–11. 33408486 10.2147/OTT.S264459 PMC7781021

[32] Tang X, Sui X, Weng L, Liu Y. SNAIL1: linking tumor metastasis to immune evasion. Front Immunol. 2021;12:724200. 34917071 10.3389/fimmu.2021.724200 PMC8669501

[33] Boyer B, Valles AM, Edme N. Induction and regulation of epithelial-mesenchymal transitions. Biochem Pharmacol. 2000;60:1091–9. 11007946 10.1016/s0006-2952(00)00427-5

[34] Gujral TS, Chan M, Peshkin L, Sorger PK, Kirschner MW, MacBeath G. A noncanonical Frizzled2 pathway regulates epithelial-mesenchymal transition and metastasis. Cell. 2014;159:844–56. 25417160 10.1016/j.cell.2014.10.032 PMC4243058

[35] Chen L, Li M, Li Q, Xu M, Zhong W. Knockdown of TRIM47 inhibits glioma cell proliferation, migration and invasion through the inactivation of Wnt/beta-catenin pathway. Mol Cell Probes. 2020;53:101623. 32603762 10.1016/j.mcp.2020.101623

[36] Huang H, Lu J, Aukhil I, Yu C, Bhut B, Marchesan J, et al. FBXO11 regulates bone development. Bone. 2023;170:116709. 36863499 10.1016/j.bone.2023.116709 PMC11008459

[37] Lee JH, Jung SM, Yang KM, Bae E, Ahn SG, Park JS, et al. A20 promotes metastasis of aggressive basal-like breast cancers through multi-monoubiquitylation of Snail1. Nat Cell Biol. 2017;19:1260–73. 28892081 10.1038/ncb3609

[38] Vinas-Castells R, Beltran M, Valls G, Gomez I, Garcia JM, Montserrat-Sentis B, et al. The hypoxia-controlled FBXL14 ubiquitin ligase targets SNAIL1 for proteasome degradation. J Biol Chem. 2010;285:3794–805. 19955572 10.1074/jbc.M109.065995 PMC2823521

