# Supplementary material for: Arginine methylation-dependent TRIM47 stability mediated by CARM1 promotes the metastasis of hepatocellular carcinoma
Source: Cell Death Discov. 2024 Nov 20;10:477. doi: 10.1038/s41420-024-02244-4 (PMC11579460; doi:10.1038/s41420-024-02244-4)
Supplement: Supplementary file 1 — Supplementary Materials [file 41420_2024_2244_MOESM1_ESM.docx]

Supplementary Materials

**Table S1.** Primer sequence for synthesizing shRNA

| Name |  | Primer sequence |
| --- | --- | --- |
| shTRIM47-1# |  | CCGGCAAGAAGTCCTGCATATCCGTCTCGAGACGGATATGCAGGACTTCTTGTTTTTG |
| shTRIM47-2# |  | CCGGGTTTGCCTATATTGTGGATTTCTCGAGAAATCCACAATATAGGCAAACTTTTTG |
| shCARM1-1# |  | CCGGCTATGGGAACTGGGACACTTTCTCGAGAAAGTGTCCCAGTTCCCATAGTTTTTG |
| shCARM1-2# |  | CCGGCGATTTCTGTTCCTTCTACAACTCGAGTTGTAGAAGGAACAGAAATCGTTTTTG |


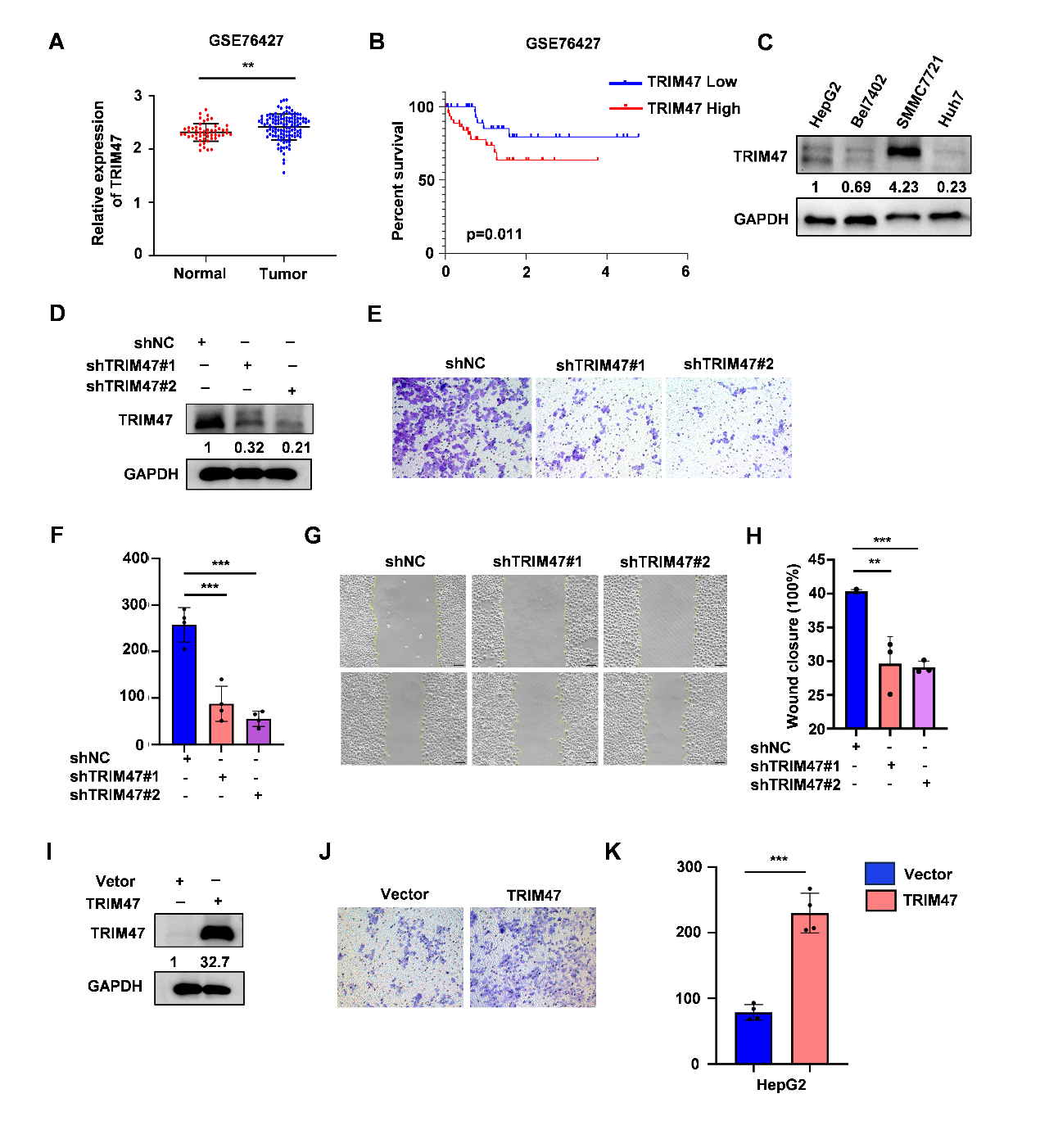


**Figure S1 A** Analysis of TRIM47 mRNA expression in HCC primary tumor tissues and normal liver tissues from GSE76427 database. **, *P* < 0.01. **B** Kaplan-Meier survival analysis of overall survival (OS) stratified by TRIM47 expression in HCC tissues from GSE76427 database. *P*=0.011. **C** Western blot analysis of TRIM47 protein levels in HepG2, Bel7402, SMMC7721 and Huh7 cell lines. **D** Western blot analysis of TRIM47 protein levels in TRIM47 stably knockdown (shTRIM47#1 and shTRIM47#2) or control (shNC) HepG2 cells. **E-F** The migration assay of HepG2 cells with TRIM47 stably knockdown (**E**). The average number of cells per field were calculated (**F**). Scale bars, 50μm. Data are shown as mean ± SD. n = 3 samples per group, four fields per sample. ***, *P* < 0.001. **G-H** Wound*-*healing assay of SMMC7721 cells with TRIM47 stably knockdown (**G**). Quantification of wound closure at the 72 h (**H**). Scale bars, 50μm. Data are shown as mean ± SD. **, *P* < 0.01, ***, *P* < 0.001. **I** Western blot analysis of TRIM47 protein levels in HepG2 cells transiently transfected with Vector or FLAG-TRIM47. **J-K** The migration assay of HepG2 cells transiently transfected with Vector or FLAG-TRIM47 (**J**). The average number of cells per field were calculated (**K**). Scale bars, 50μm. Data are shown as mean ± SD. n = 3 samples per group, four fields per sample. ***, *P* < 0.001.


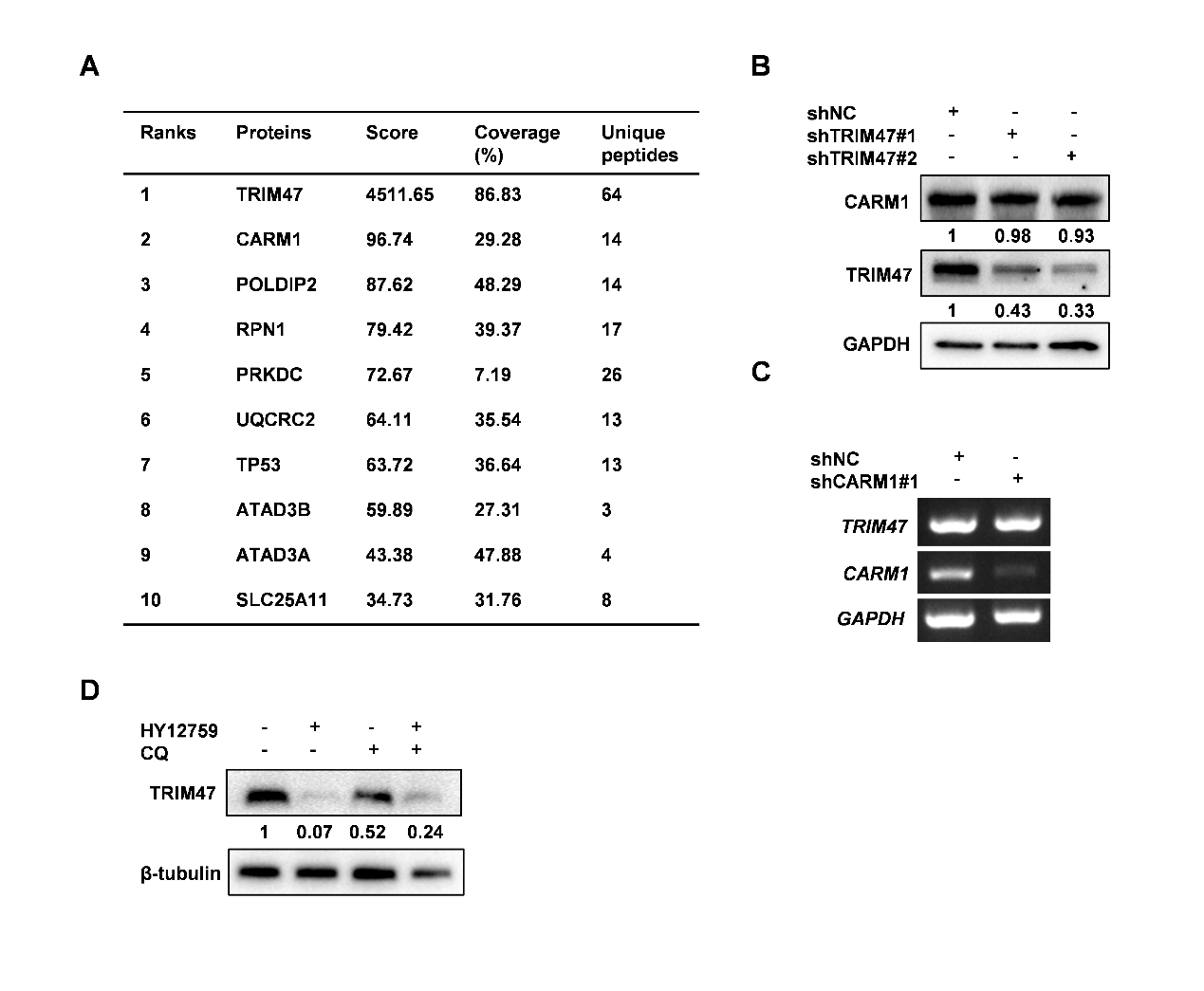


**Figure S2 A** The top 10 TRIM47-interacting proteins based on spectral matching score by mass spectrometry. **B** Western blot analysis of CARM1 protein levels in TRIM47 stably knockdown SMMC7721 cells. **C** RT-PCR analysis of TRIM47 mRNA expression in CARM1 stably knockdown SMMC7721 cells. **D** Western blot analysis of TRIM47 protein levels in SMMC7721 cells treated with 10μM HY12759 and 50 μM CQ for 8 h.


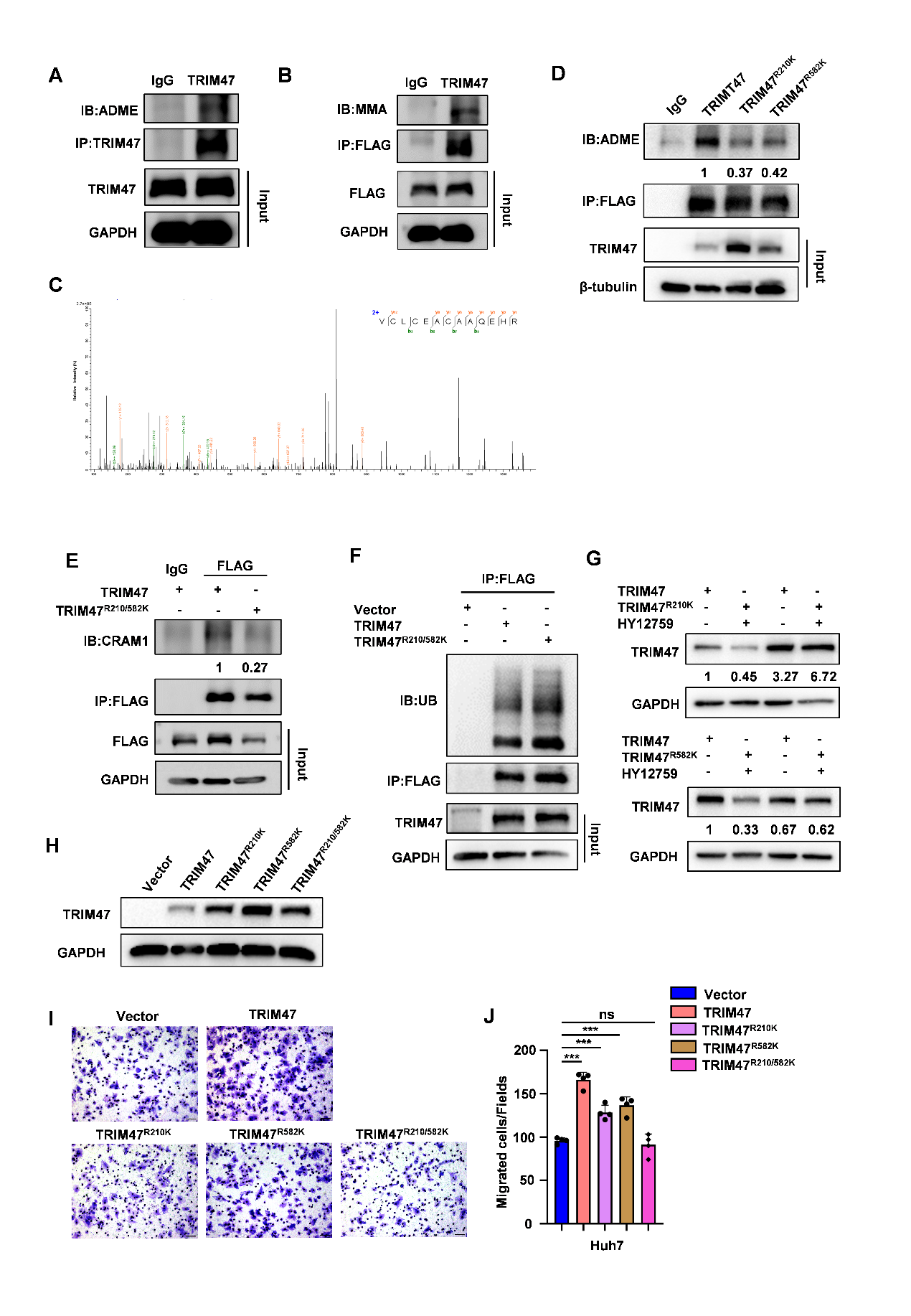


**Figure S3 A** SMMC7721 cell extracts were subjected to immunoprecipitated with anti-TRIM47 antibody or control IgG. Arginine methylation (ADME) of immunopurified TRIM47 was detected by western blot. **B** SMMC7721 cells were transfected with FLAG-TRIM47 for 48 h. Cell extracts were subjected to immunoprecipitated with anti-FLAG antibody or control IgG. Arginine methylation (MMA) of immunopurified TRIM47 was detected by western blot. **C** SMMC7721 cells were transfected with FLAG-TRIM47 for 48 h. Cell extracts were subjected to immunoprecipitated with anti-FLAG antibody or control IgG. Arginine methylation of immunopurified TRIM47 was detected by western blot. R210 was identified via mass spectrometry. **D** SMMC7721 cells were transiently transfected with FLAG-TRIM47, FLAG-TRIM47^R210K^ or FLAG-TRIM47^R582K^ for 48 h. Cell extracts were subjected to immunoprecipitated with anti-FLAG antibody. Arginine methylation of immunopurified TRIM47 was detected by western blot. **E** SMMC7721 cells were transfected with FLAG-TRIM47 or FLAG-TRIM47^R210/582K^ for 48h. Cell extracts were immunoprecipitated with anti-FLAG antibody. The immunoprecipitates were detected with anti-CARM1 and anti-FLAG antibodies. **F** SMMC7721 cells were co-transfected with FLAG-TRIM47 or FLAG-TRIM47^R210/582K^ and HA-ubiquitin plasmids for 48h. Cell extracts were immunoprecipitated with anti-FLAG antibody. The immunoprecipitates were detected by with anti-ubiquitin and anti-FLAG antibodies. **G** SMMC7721 cells were transfected with FLAG-TRIM47, FLAG-TRIM47^R210K^ (upper) or FLAG-TRIM47^R582K^ (bottom) for 48h and treated with HY12759 for another 12 h. Western blot analysis of TRIM47 protein levels. **H** Western blot analysis of TRIM47 protein levels in SMMC7721 cells transiently transfected with Vector, FLAG-TRIM47, FLAG-TRIM47^R210K^, FLAG-TRIM47^R582K^ or FLAG-TRIM47^R210/582K^. **I-J** The migration assay of Huh7 cells transiently transfected with Vector, FLAG-TRIM47, FLAG-TRIM47^R210K^, FLAG-TRIM47^R582K^ or FLAG-TRIM47^R210/582K^ (**I**). The average number of cells per field were calculated (**J**). Scale bars, 50μm. Data shown as mean ± SD. n = 3 samples per group, four fields per sample. ***, *P* < 0.001, ns, no significance*.*


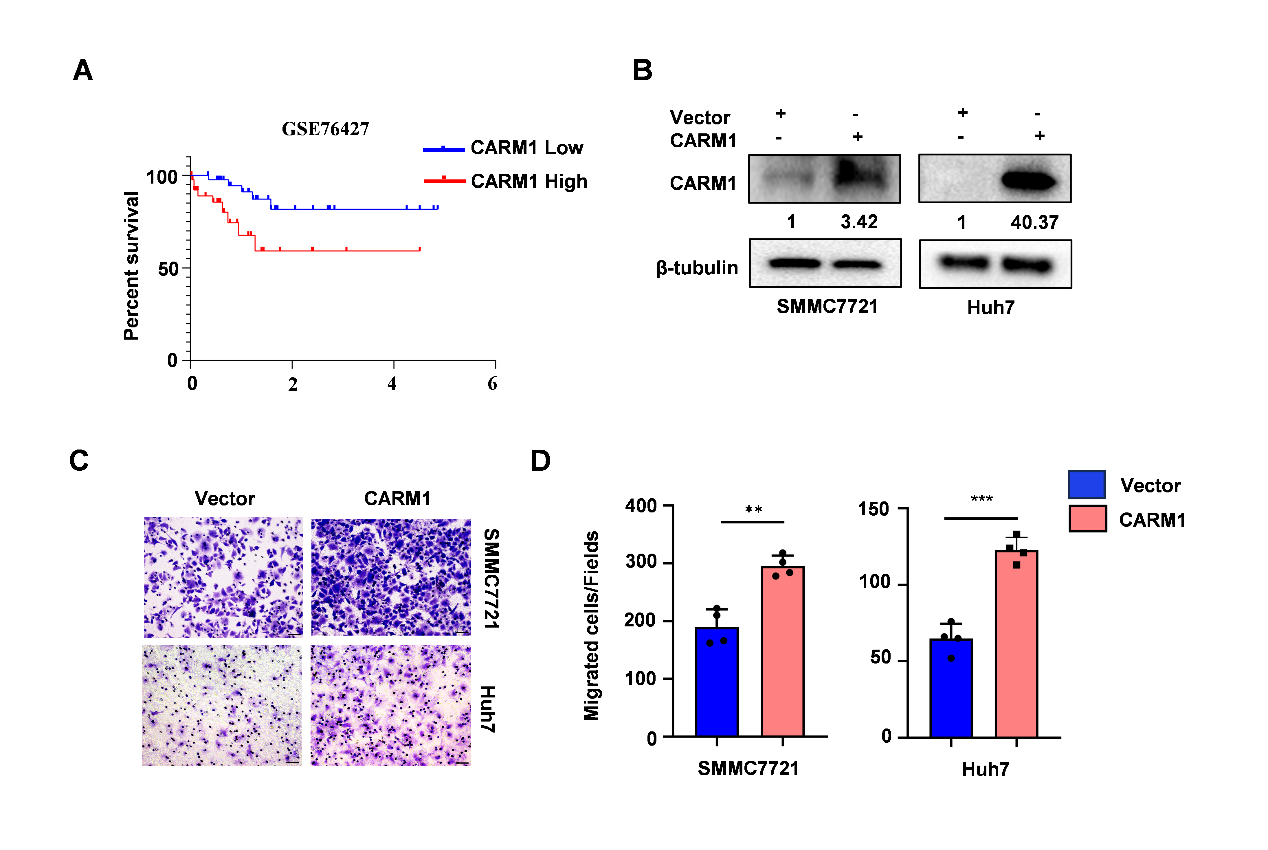


**Figure S4 A** Kaplan-Meier survival analysis of overall survival (OS) stratified by CARM1 expression in HCC tissues from GSE76427 database. **B** Western blot analysis of CARM1 protein levels in SMMC7721 and Huh7 cells transfected with Vector or FLAG-CARM1. **C-D** The migration assay of SMMC7721 and Huh7 cells transfected with Vector or FLAG-CARM1 (**C**). The average number of cells per field were calculated (**D**). Scale bars, 50μm. Data shown as mean ± SD. n = 3 samples per group, four fields per sample. **, *P* < 0.01, ***, *P* < 0.001.


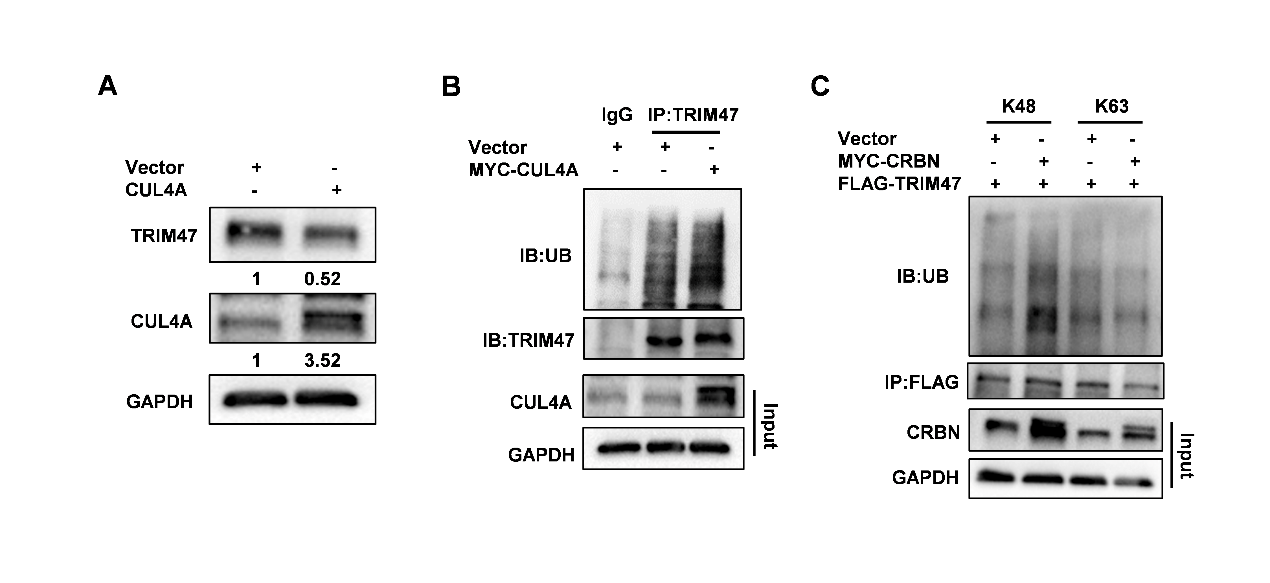


**Figure S5 A** Western blot analysis of TRIM47 expression in SMMC7721 cells transfected with MYC-CUL4A for 48 h. **B** SMMC7721 cells were co-transfected with MYC-CUL4A and HA-ubiquitin plasmids for 48h. Total cell extracts were immunoprecipitated with anti-TRIM47 antibody. The immunoprecipitates were detected by with anti-ubiquitin and anti-TRIM47 antibodies. **C** Immunoprecipitation analysis of TRIM47 ubiquitination in SMMC7721 cells co-transfected with MYC-CRBN and HA–ubiquitin (K48) or HA–ubiquitin (K63).


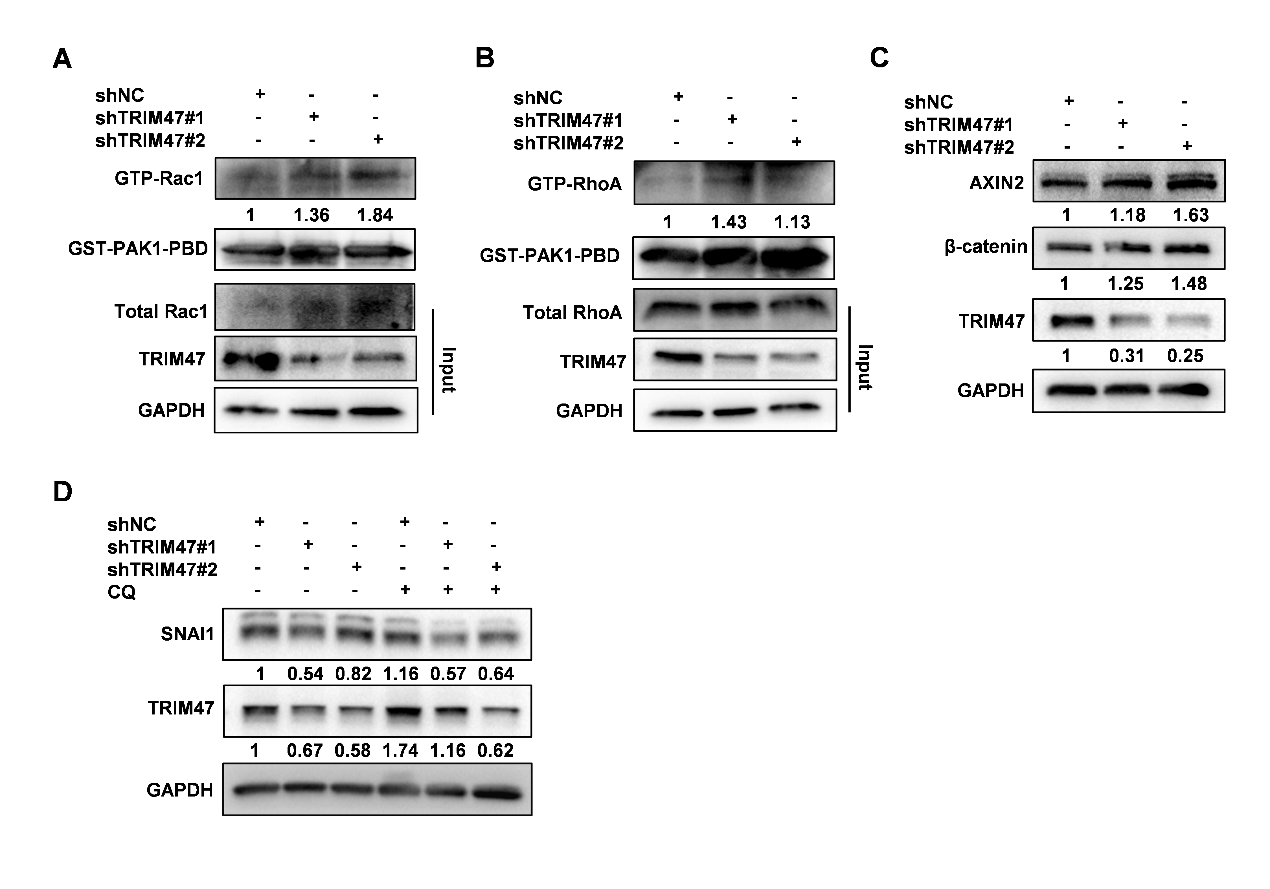


**Figure S6** **A** Guanosine-5'-triphosphate (GTP) -bound and total forms of Rac1 protein levels in TRIM47 stably knockdown (shTRIM47#1 and shTRIM47#2) or control (shNC) SMMC7721 cells. **B** Guanosine-5'-triphosphate (GTP) -bound and total forms of RhoA protein levels in TRIM47 stably knockdown (shTRIM47#1 and shTRIM47#2) or control (shNC) SMMC7721 cells. Quantification of RhoA activity was performed by normalizing GTP-RhoA to Total RhoA. **C** Western blot analysis of AXIN2 and β-catenin in TRIM47 stably knockdown SMMC7721 cells. **D** Western blot analysis of SNAI1 protein levels in TRIM47 stably knockdown (shTRIM47#1 and shTRIM47#2) or control (shNC) SMMC7721 cells treated with 50 μM CQ for 6 h.
