# Supplementary material for: Arginine methylation-dependent TRIM47 stability mediated by CARM1 promotes the metastasis of hepatocellular carcinoma
Source: Cell Death Discov. 2024 Nov 20;10:477. doi: 10.1038/s41420-024-02244-4 (PMC11579460; doi:10.1038/s41420-024-02244-4)

**Original image for Figure 1**


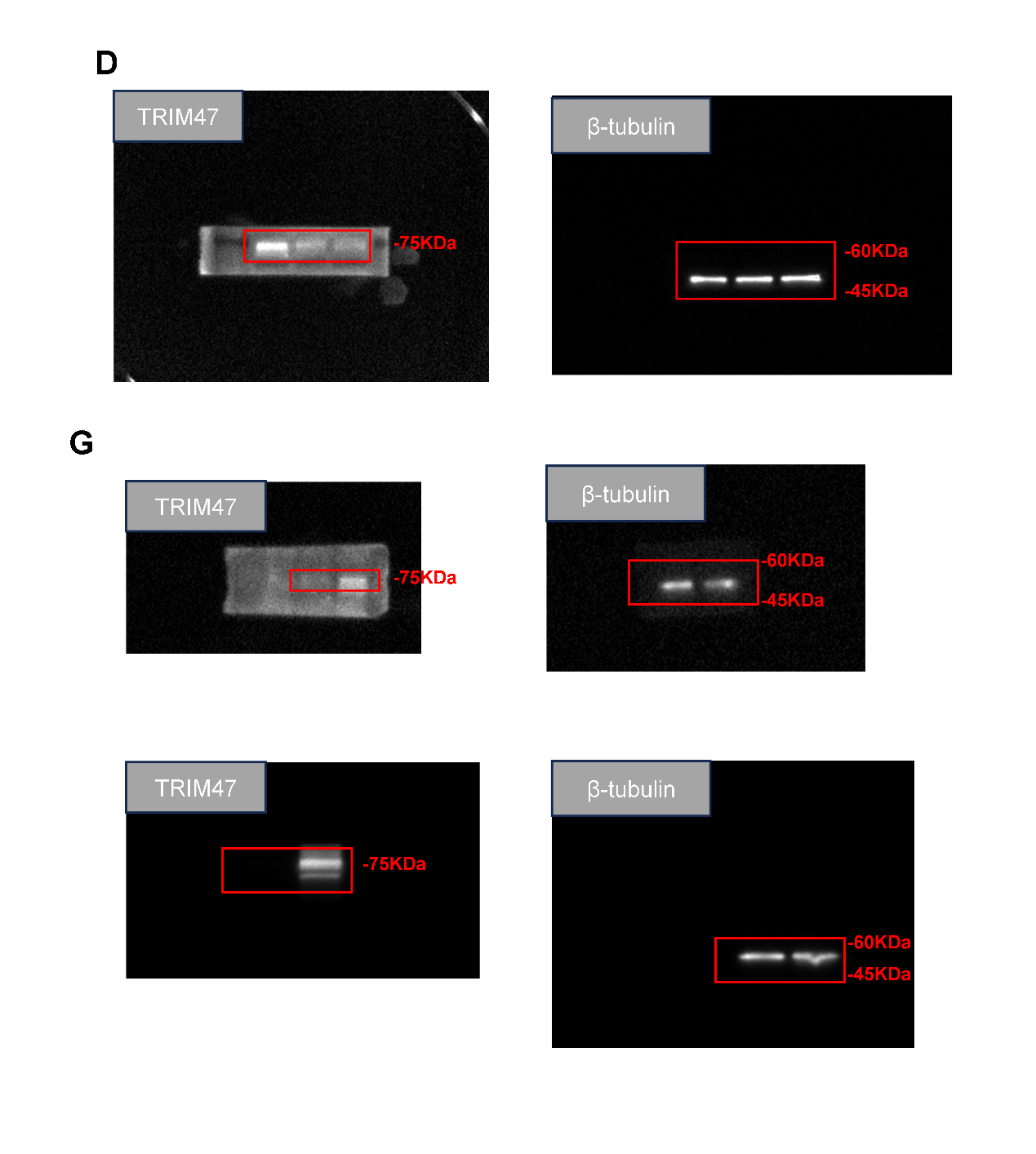


**Original image for Figure 2**


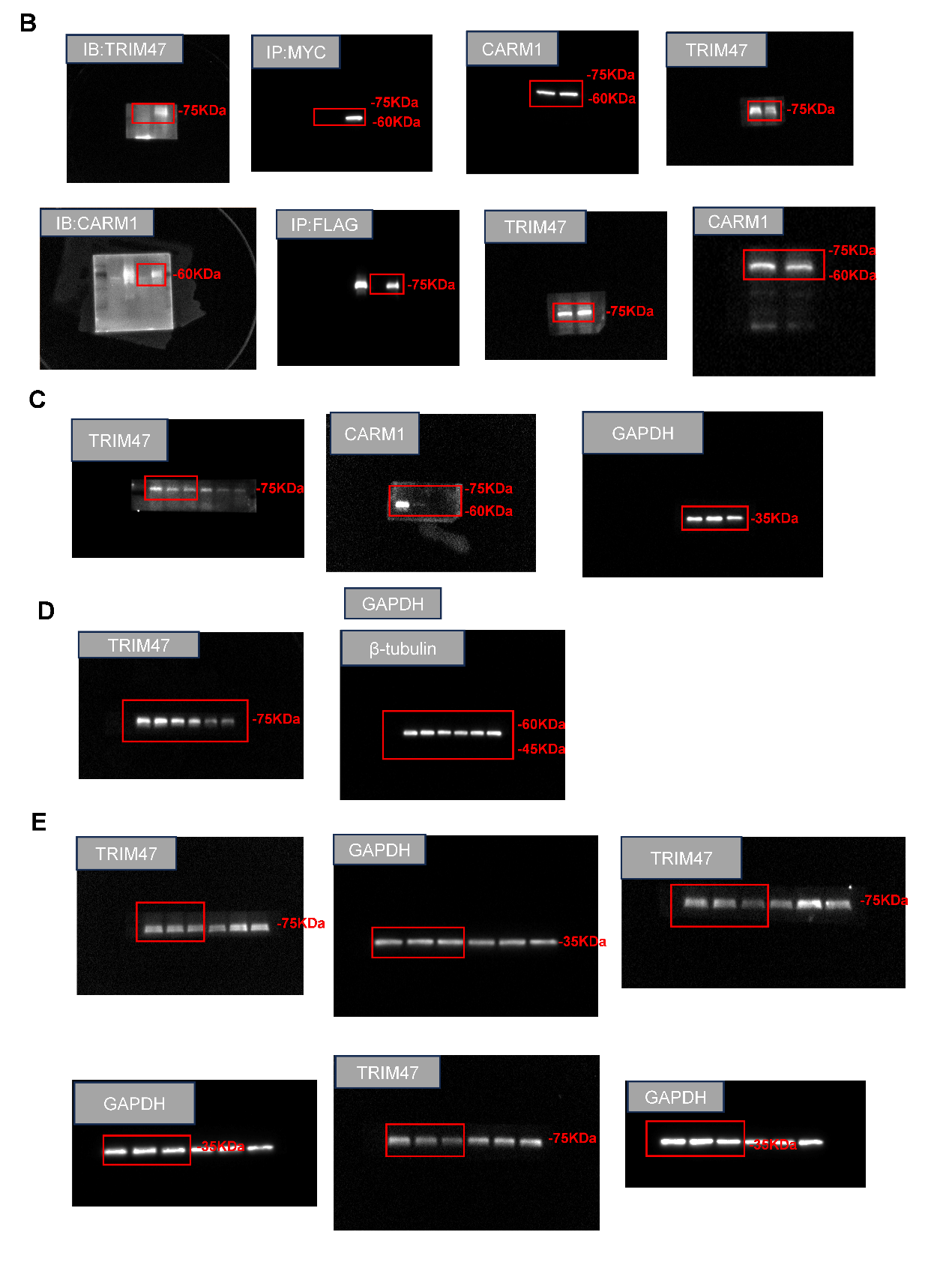


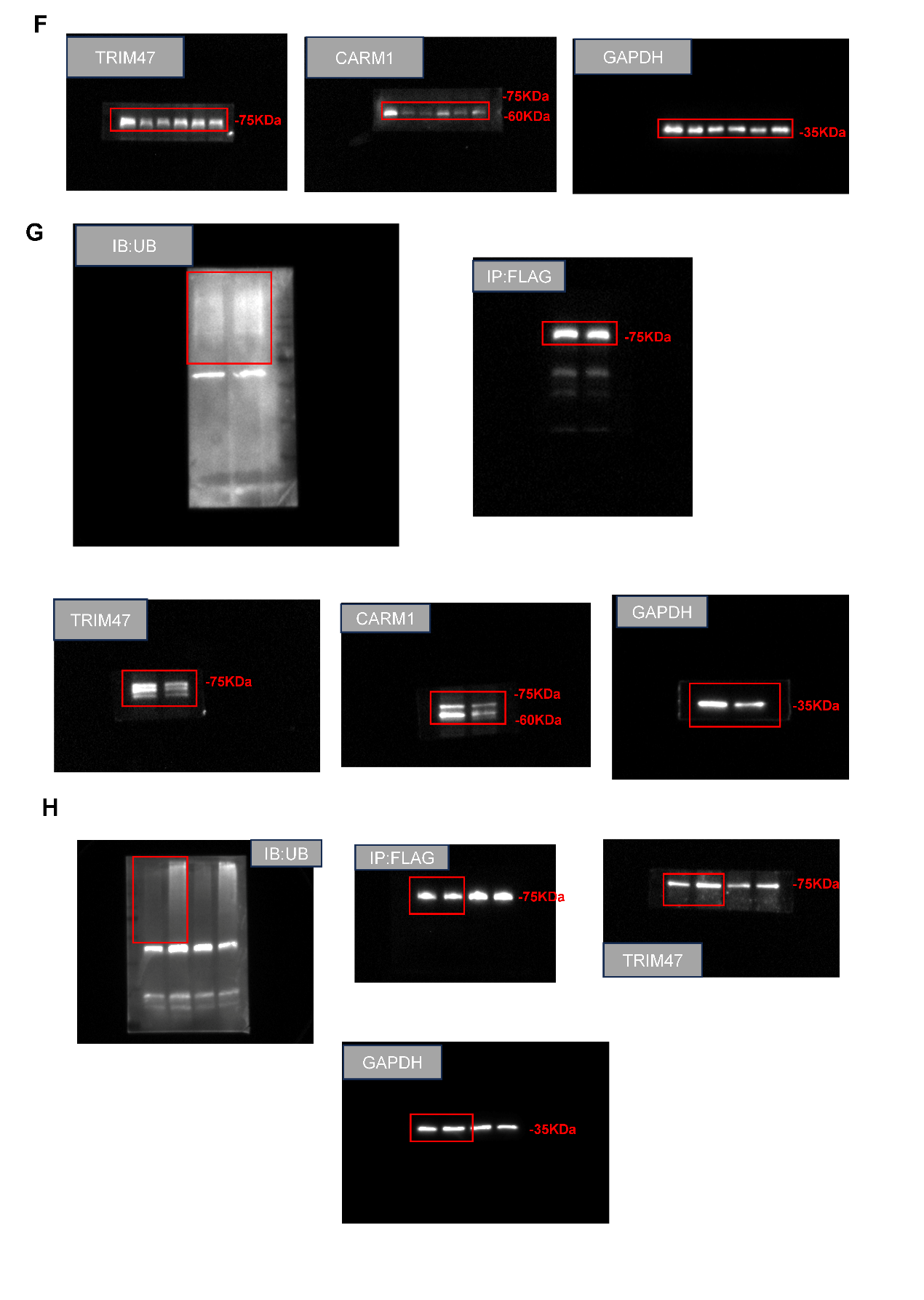


**Original image for Figure 3**


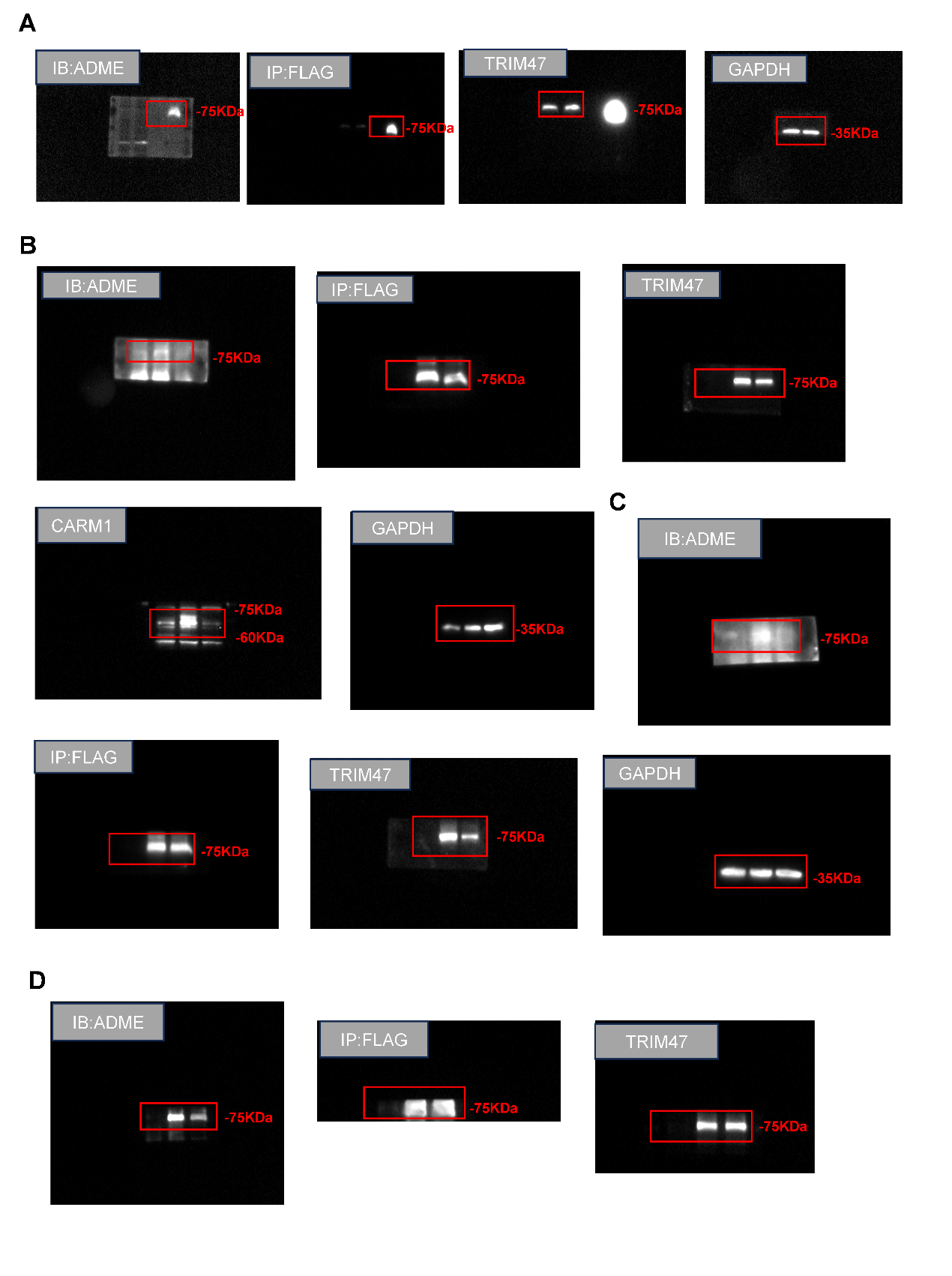


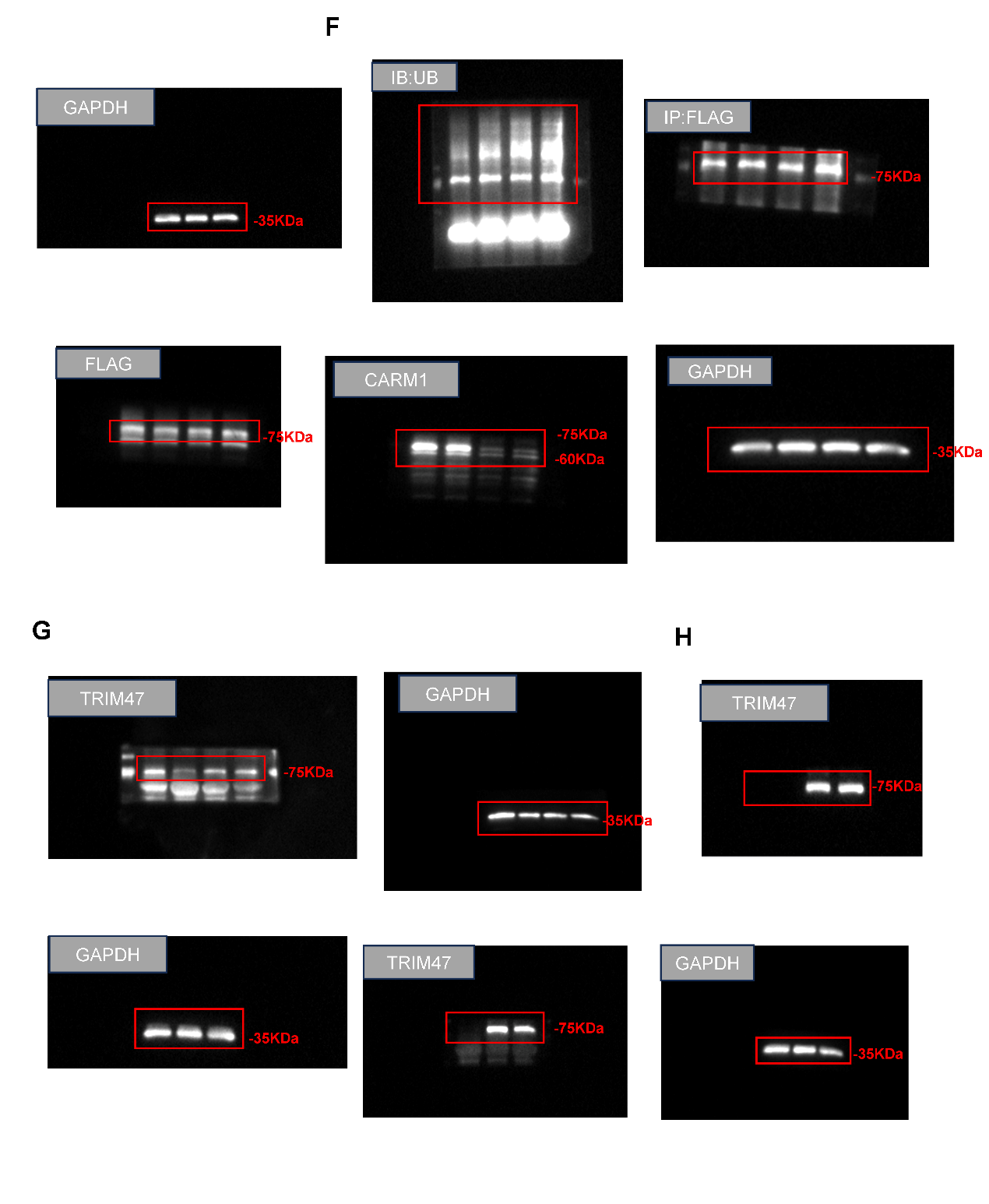


**Original image for Figure 4**


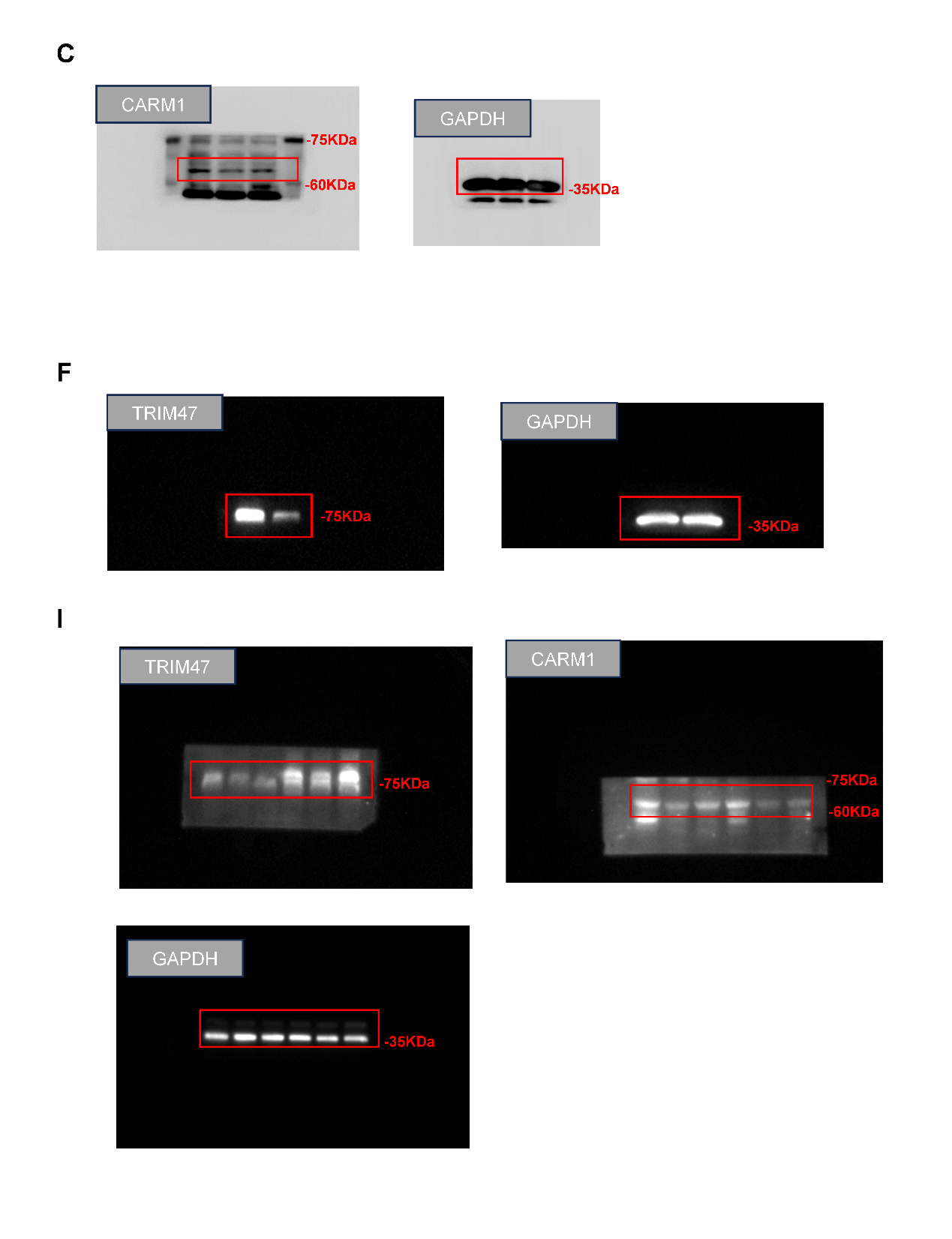


**Original image for Figure 5**


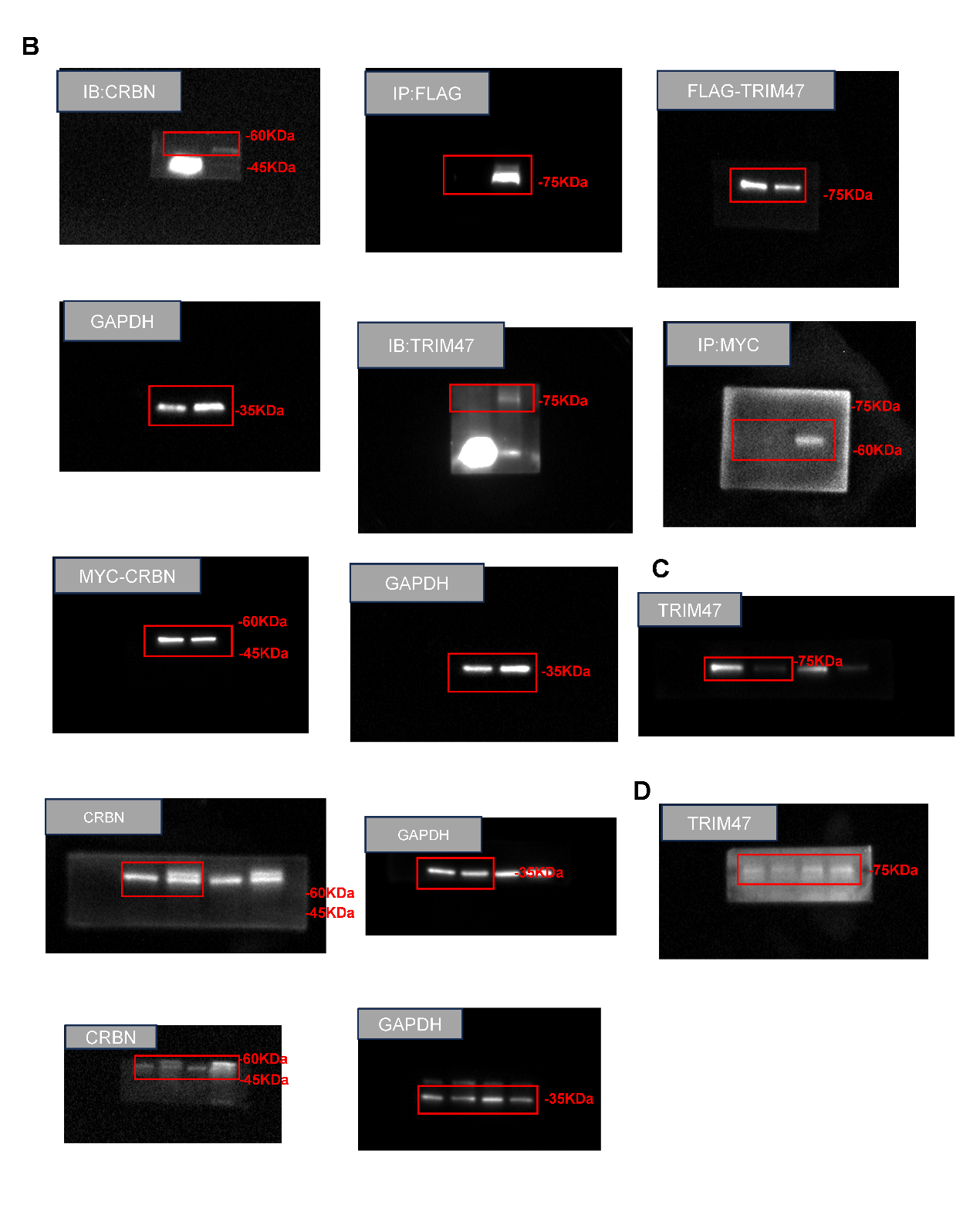

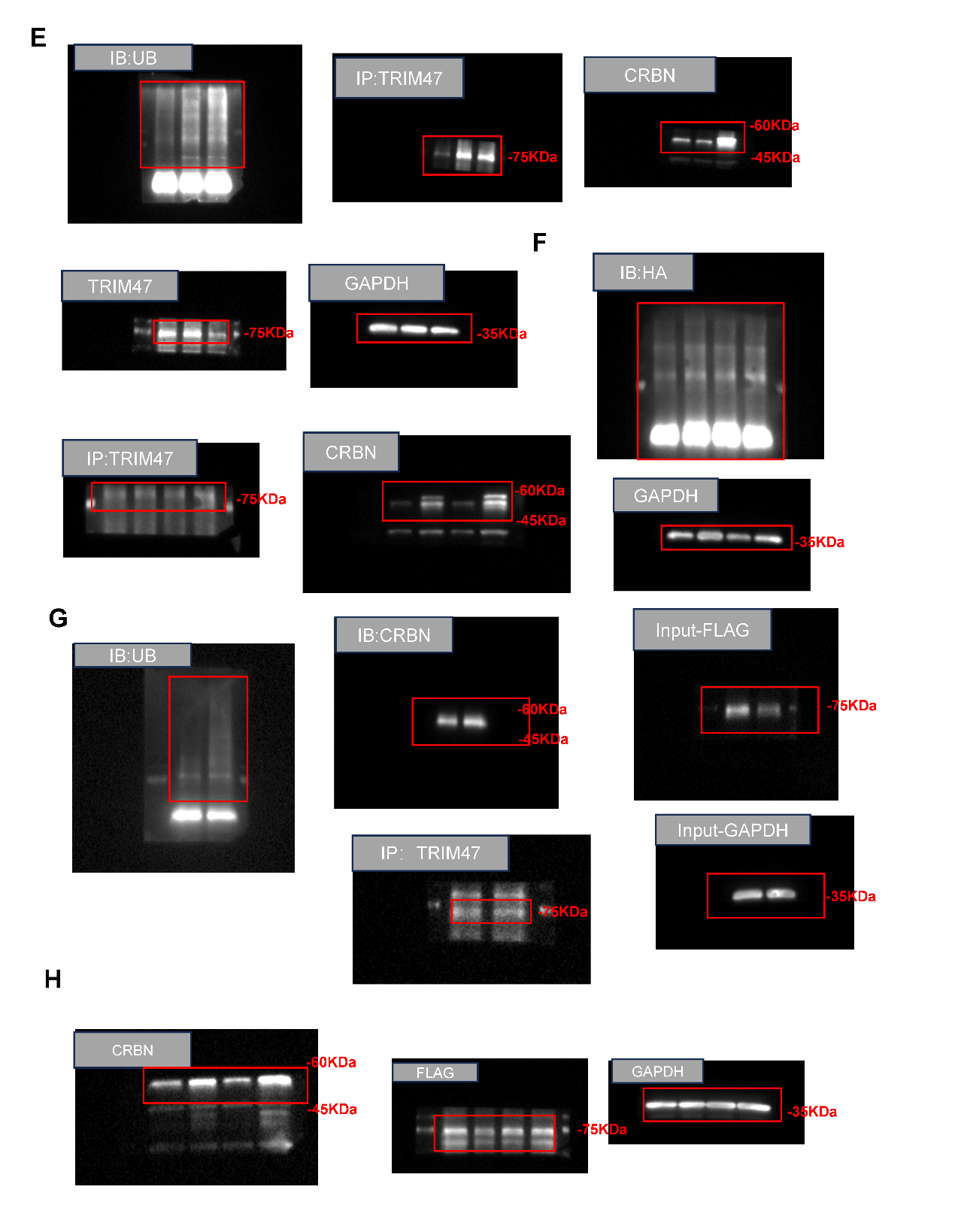


**Original image for Figure 6**


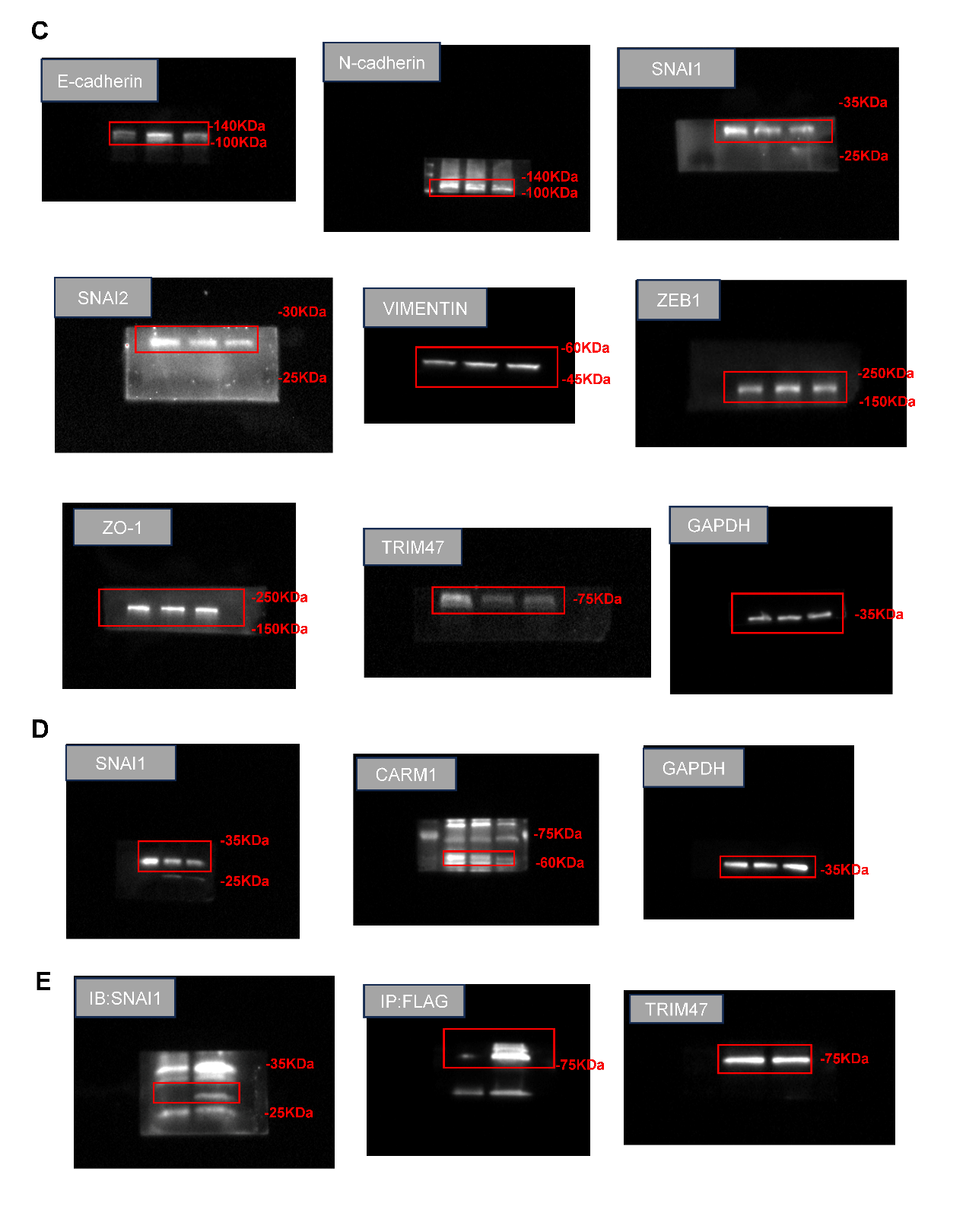


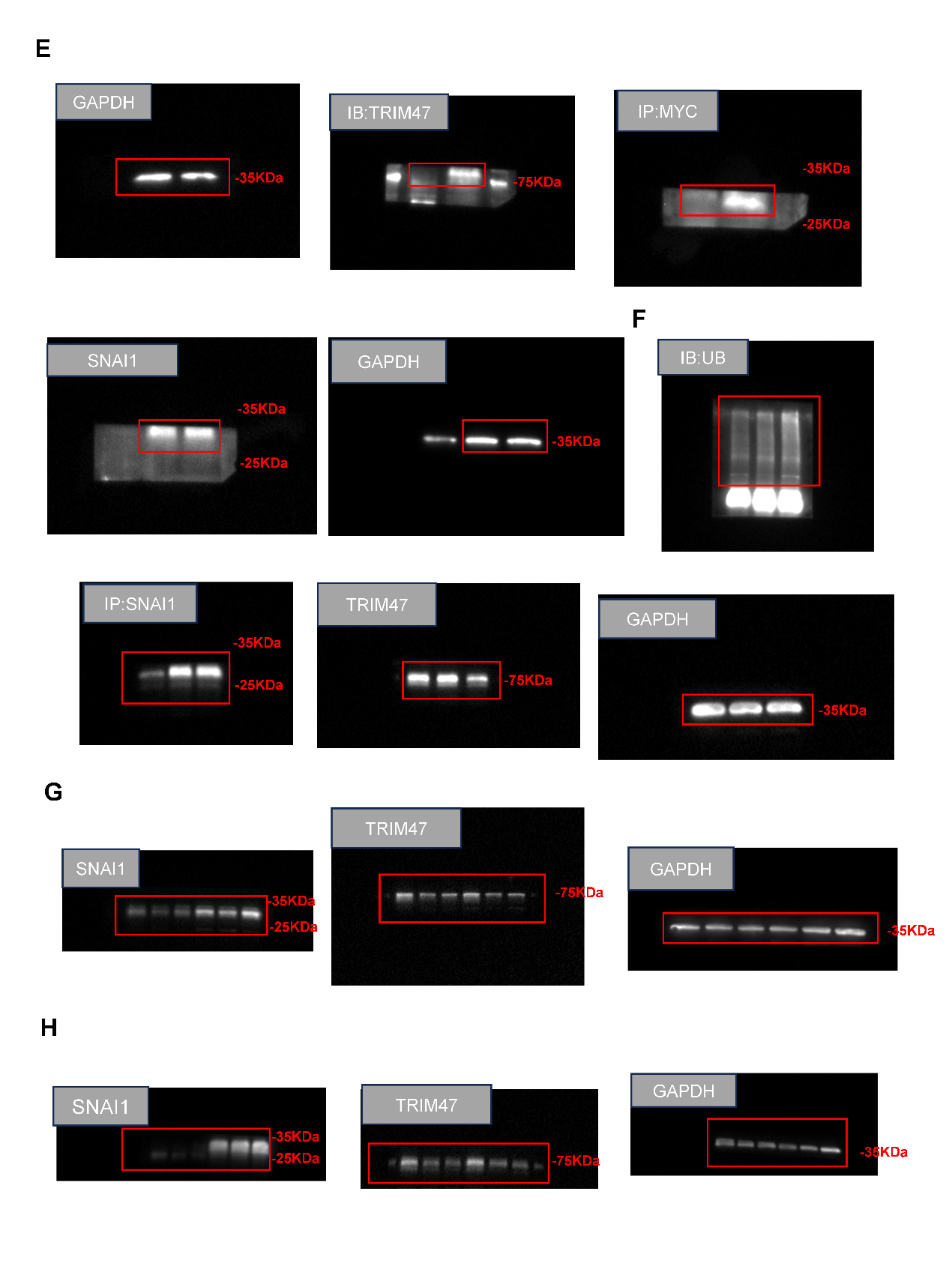


**Original image for Figure S1**


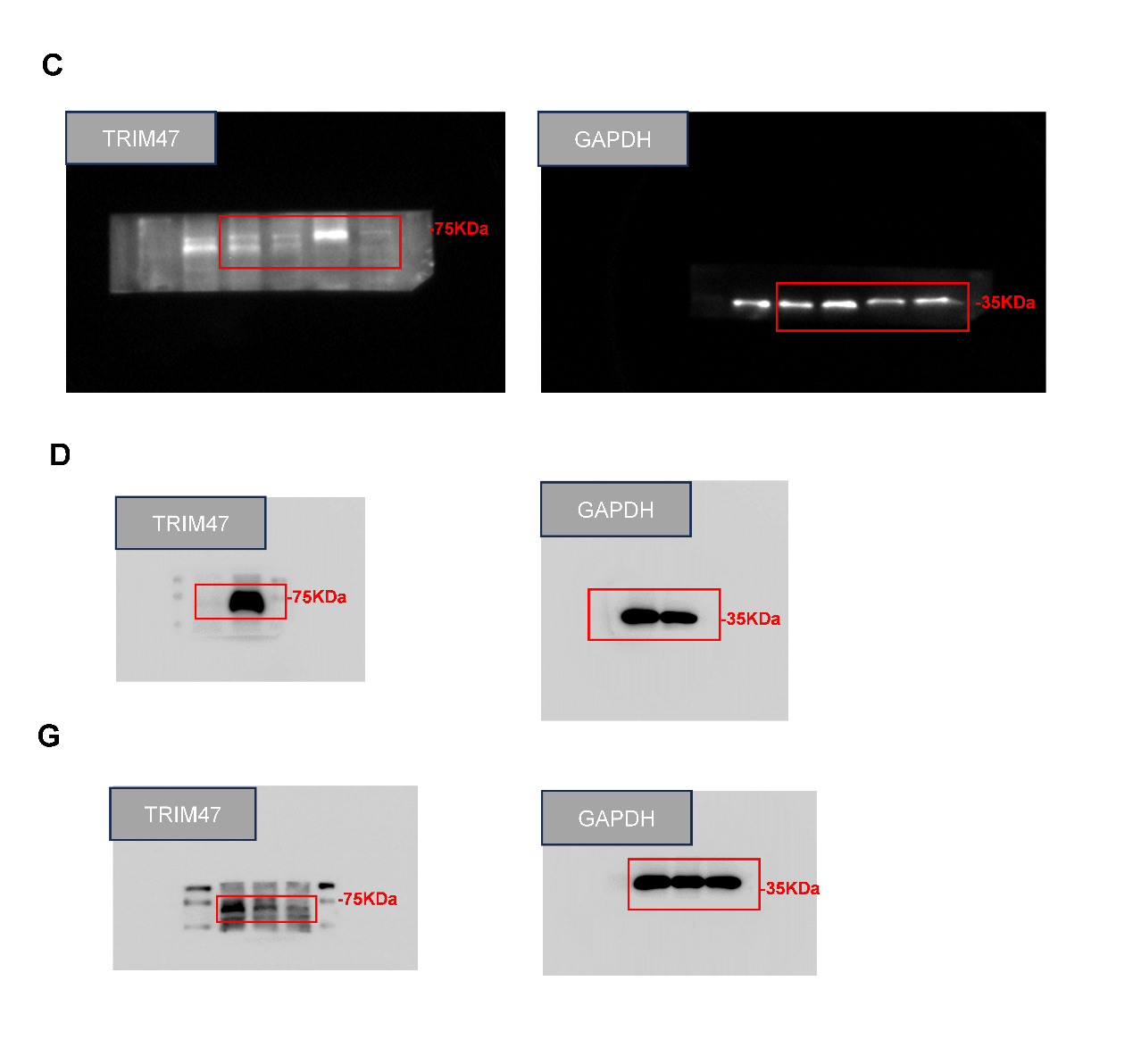


**Original image for Figure S2**


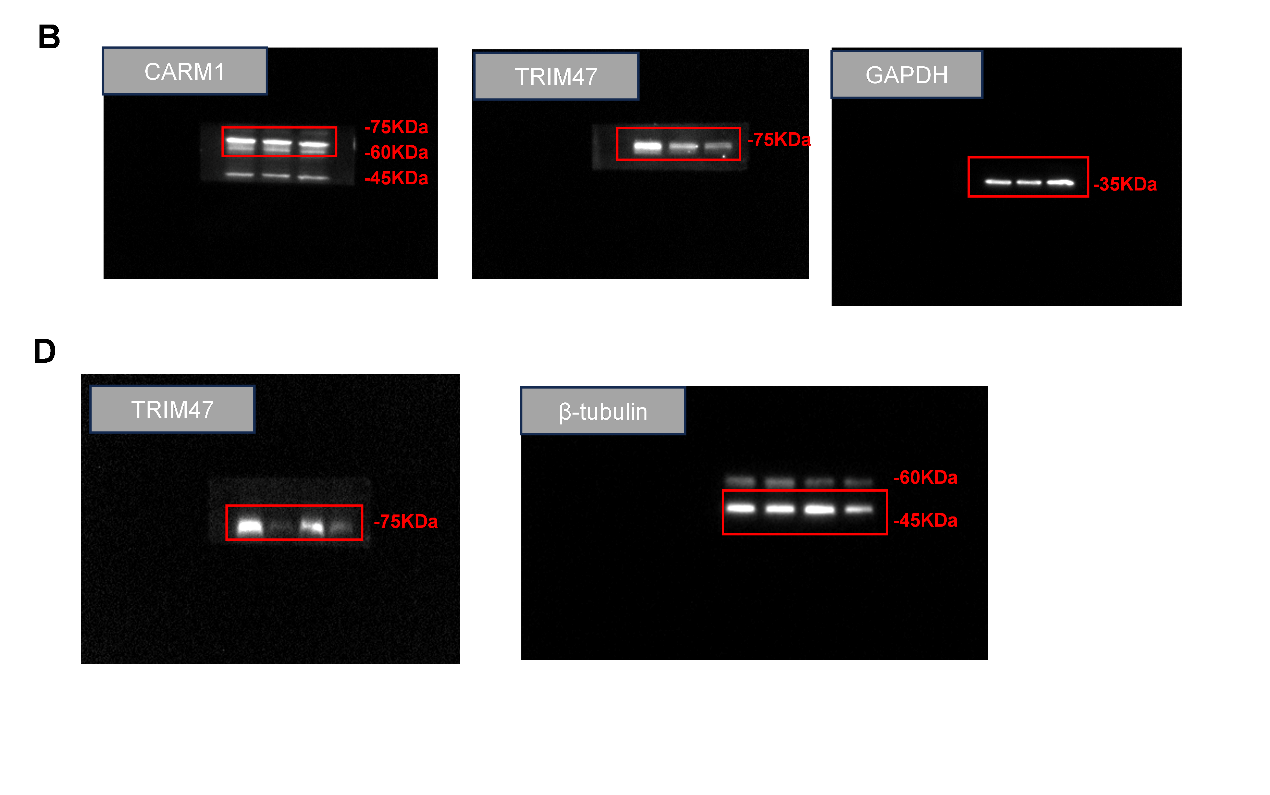


**Original image for Figure S3**


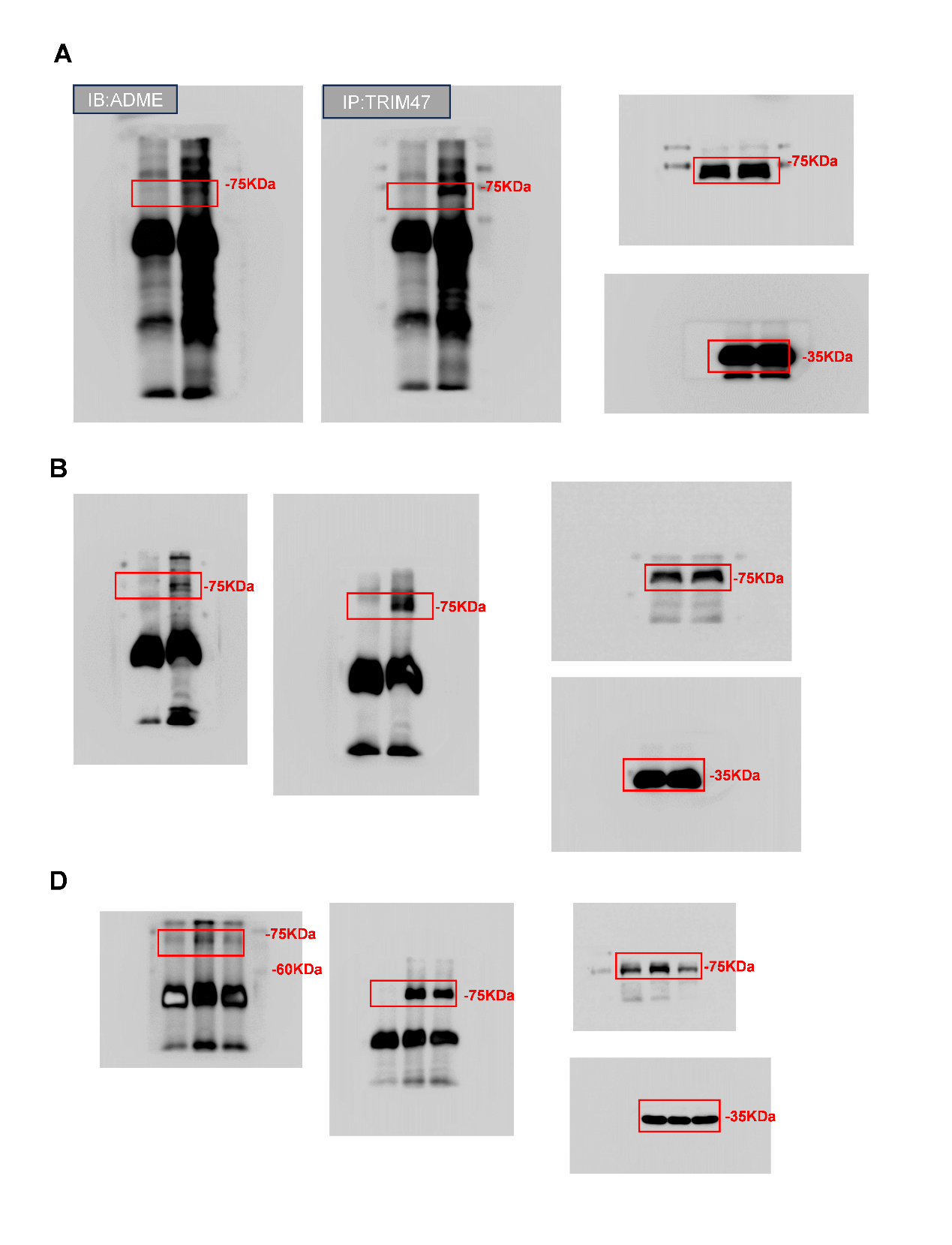

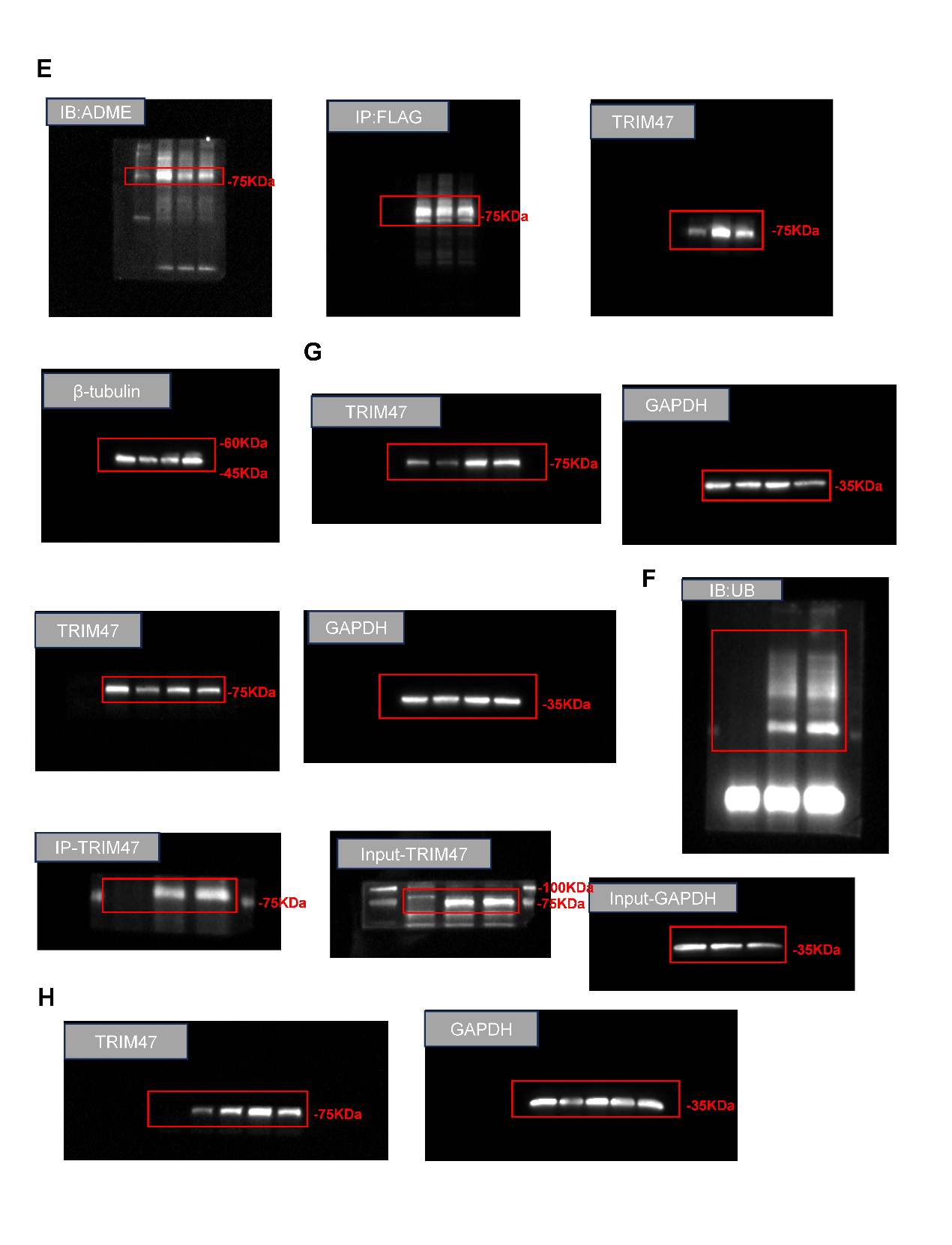


**Original image for Figure S4**


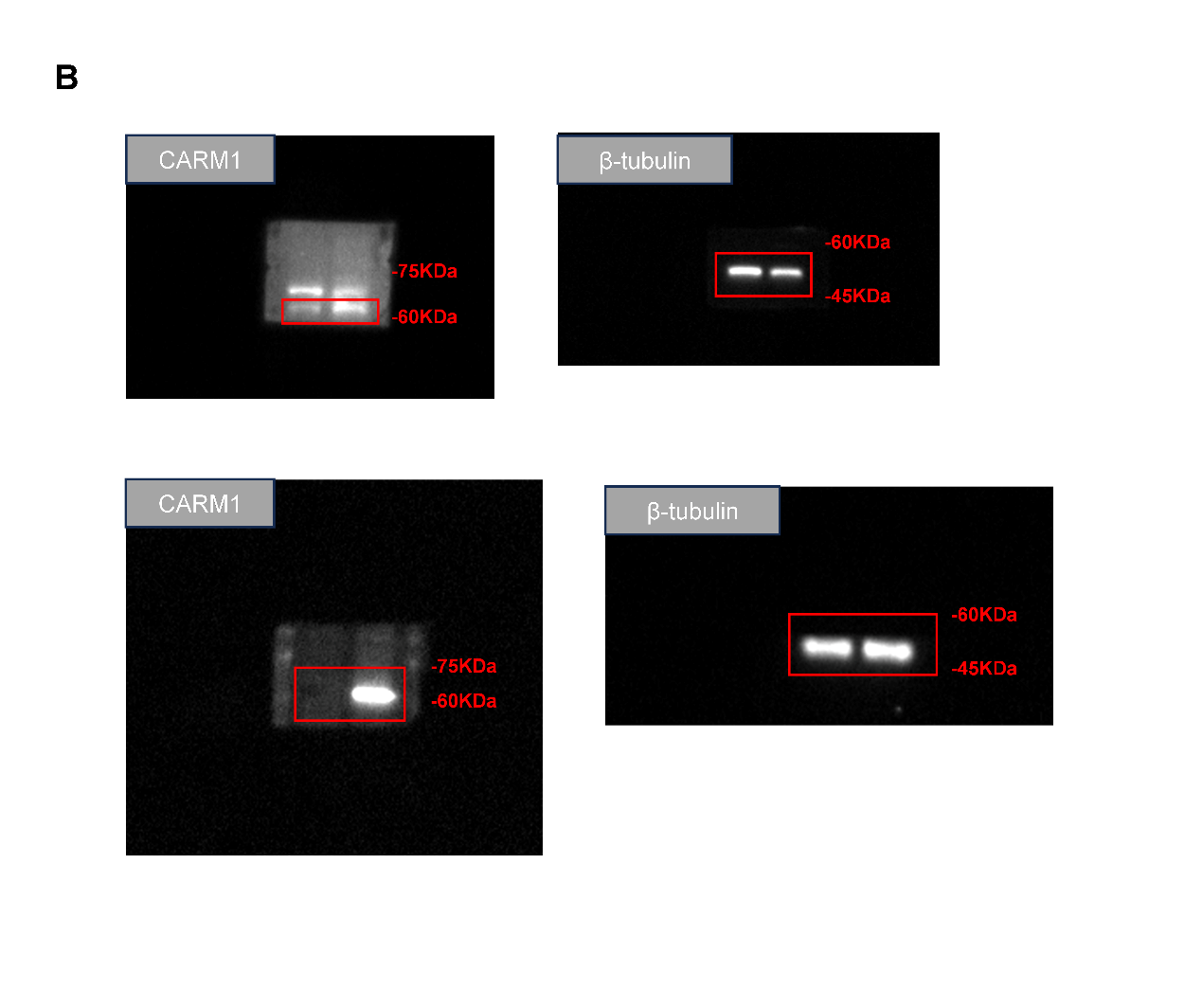


**Original image for Figure S5**


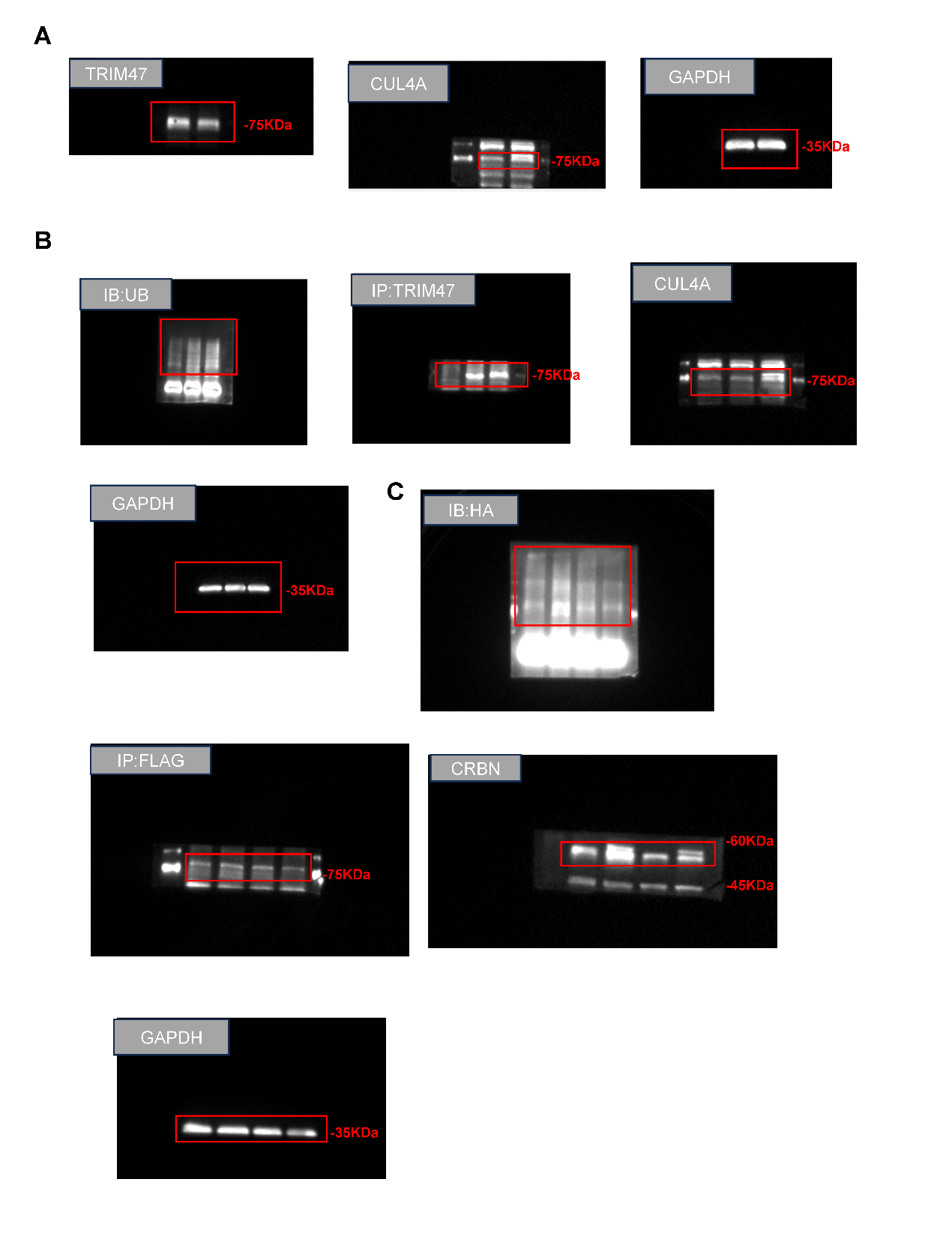


**Original image for Figure S6**


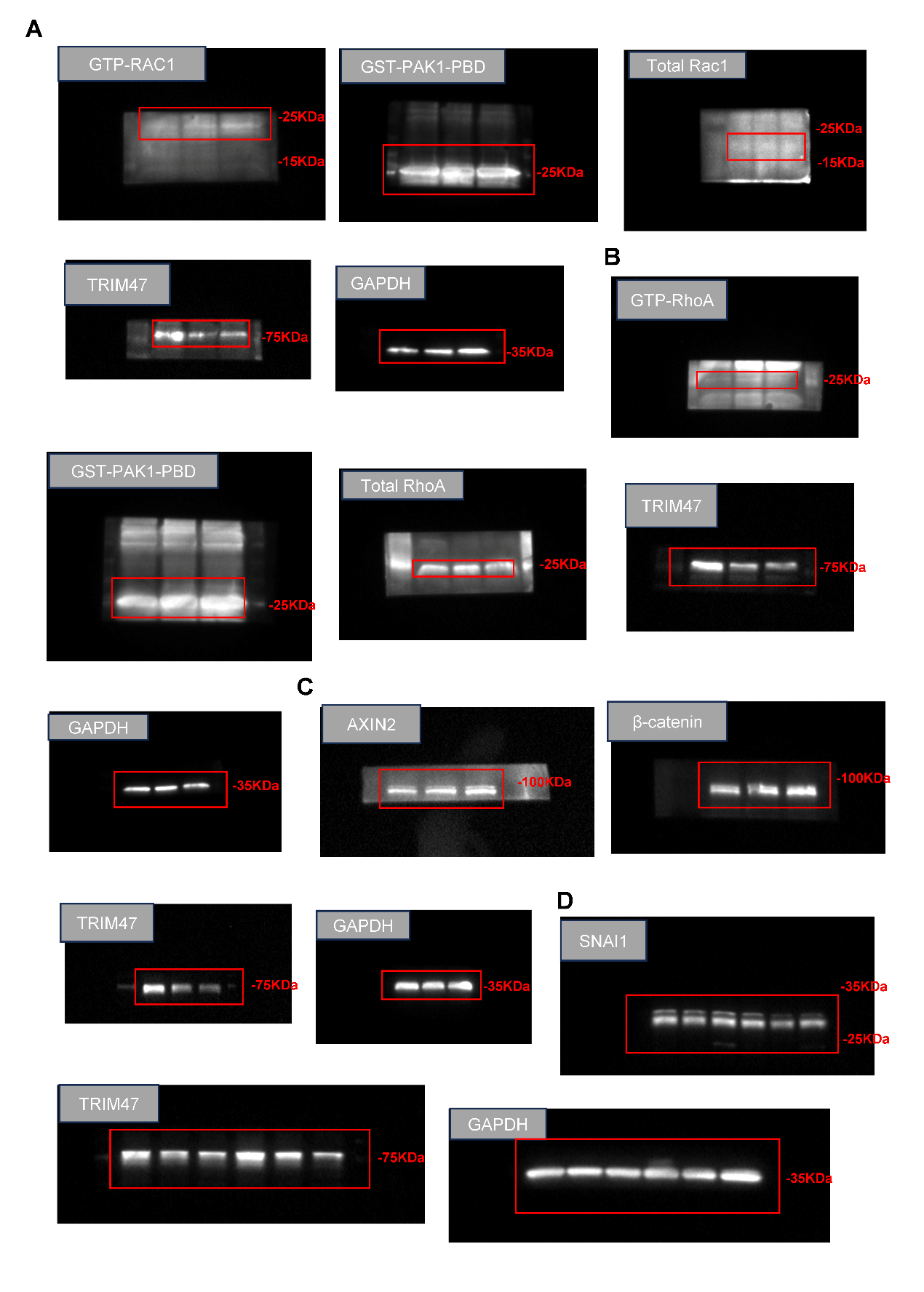

Supplement: Supplementary file 2 — Original image for western blot [file 41420_2024_2244_MOESM2_ESM.docx]
